# Effects of Ramadan Observance on Dietary Intake and Body Composition of Adolescent Athletes: Systematic Review and Meta-Analysis

**DOI:** 10.3390/nu12061574

**Published:** 2020-05-28

**Authors:** Khaled Trabelsi, Achraf Ammar, Omar Boukhris, Jordan M Glenn, Nick Bott, Stephen R. Stannard, Florian A. Engel, Billy Sperlich, Sergio Garbarino, Nicola L. Bragazzi, Roy J. Shephard, Hamdi Chtourou

**Affiliations:** 1 Institut Supérieur du Sport et de l’éducation physique de Sfax, Université de Sfax, Sfax 3000, Tunisie; trabelsikhaled@gmail.com (K.T.); omarboukhris24@yahoo.com (O.B.); h_chtourou@yahoo.fr (H.C.); 2 Research Laboratory: Education, Motricité, Sport et Santé, EM2S, LR19JS01, High Institute of Sport and Physical Education of Sfax, University of Sfax, Sfax 3000, Tunisia; 3 Institute of Sport Science, Otto von Guericke University Magdeburg, 39104 Magdeburg, Germany; ammar.achraf@ymail.com; 4 Activité Physique: Sport et Santé, UR18JS01, Observatoire National du Sport, Tunis 1003, Tunisie; 5 Exercise Science Research Center, Department of Health, Human Performance and Recreation, University of Arkansas, Fayetteville, AR 72701, USA; jordan@neurotrack.com; 6 Neurotrack Technologies, 399 Bradford St, Redwood City, CA 94063, USA; nick@neurotrack.com; 7 Clinical Excellence Research Center, Department of Medicine, Stanford University School of Medicine, Stanford, CA 94305, USA; 8 School of Sport and Exercise, College of Health, Massey University, Palmerston North 4442, New Zealand; sstannard@gmail.com; 9 Institute of Sport and Sport Science, Department of Movement and Training Science, Heidelberg University, 69120 Heidelberg, Germany; florian.engel@issw.uni-heidelberg.de; 10 Institute of Sport Science, University of Würzburg, 97082 Würzburg, Germany; billy.sperlich@uni-wuerzburg.de; 11 Department of Neuroscience, Rehabilitation, Ophthalmology, Genetics, Maternal and Child, Health (DINOGMI), University of Genoa, 16132 Genoa, Italy; sgarbarino.neuro@gmail.com; 12 Department of Health Sciences (DISSAL), Postgraduate School of Public Health, University of Genoa, 16132 Genoa, Italy; 13 Laboratory for Industrial and Applied Mathematics (LIAM), Department of Mathematics and Statistics, York University, 4700 Keele Street, Toronto, ON M3J 1P3, Canada; 14 Faculty of Kinesiology and Physical Education, University of Toronto, Toronto, ON M5S 1A1, Canada; royjshep@shaw.ca

**Keywords:** athletes, adolescent, energy intake, fat mass, lean mass, Ramadan, systematic review and meta-analysis

## Abstract

To evaluate the effects of Ramadan observance on dietary intake, body mass and body composition of adolescent athletes (design: systematic review and meta-analysis; data sources: PubMed and Web of Science; eligibility criteria for selecting studies: single-group, pre-post, with or without control-group studies, conducted in athletes aged <19 years, training at least 3 times/week, and published in any language before 12 February 2020). Studies assessing body mass and/or body composition and/or dietary intake were deemed eligible. The methodological quality was assessed using ‘QualSyst’. Of the twelve selected articles evaluating body mass and/or body composition, one was of strong quality and eleven were rated as moderate. Ten articles evaluated dietary intake; four were rated as strong and the remaining moderate in quality. Continuation of training during Ramadan did not change body mass from before to the first week (trivial effect size (ES) = −0.011, *p* = 0.899) or from before to the fourth week of Ramadan (trivial ES = 0.069, *p* = 0.277). Additionally, Ramadan observance did not change body fat content from before to the first week (trivial ES = −0.005, *p* = 0.947) and from before to the fourth week of Ramadan (trivial ES = -0.057, *p* = 0.947). Lean body mass remained unchanged from before to the fourth week of Ramadan (trivial ES = −0.025, *p* = 0.876). Dietary data showed the intake of energy (small ES = -0.272, *p* = 0.182), fat (trivial ES = 0.044, *p* = 0.842), protein (trivial ES = 0.069, *p* = 0.720), carbohydrate (trivial ES = 0.075, *p* = 0.606) and water (trivial ES = −0.115, *p* = 0.624) remained essentially unchanged during as compared to before Ramadan. Continued training of adolescent athletes at least three times/week during Ramadan observance has no effect on body mass, body composition or dietary intake.

## 1. Introduction

The main goal of athletes is to improve performance in order to maximize success during competition. Body composition and body mass, manipulatable by training and diet, are two of many factors (e.g., physical, physiological, genetic, and psychological) that contribute to athletic success [1,2]. As a result, measures of body composition, including body fat mass and lean mass, are among the most commonly used predictors of performance [3]. In fact, studies show that in most sports, low values of fat mass and high values of lean mass are associated with better performance outcomes [4,5,6,7,8,9]. 

Nutritional strategies can play a significant role in manipulating body composition (e.g., skeletal muscle hypertrophy, decreasing fat mass), ultimately influencing athletic performance [10]. Optimizing intake, type, quantity and timing of specific foods, fluids, and supplements are widely accepted as means of enhancing athletic performance and recovery from exhaustive exercise [11,12]. Additionally, nutrition plays a very important role in maintaining the general health of athletes (e.g., respiratory and gut health, mucosal immunity) [13].

During the religious month of Ramadan (a period of 29 or 30 days), Muslim pubescent athletes abstain from eating, drinking and sexual activities from sunrise to sunset [14,15]. If Ramadan falls during the summer, the requirement to eat only overnight may lead to several changes in sleep patterns and eating schedules, potentially impacting dietary intake, body composition and physical performance [14,15]. In an attempt to maintain the proper protein intake to support muscle tissue and to sustain blood glucose levels during daylight hours, eating patterns may be deliberately modified to accommodate late-night training. As a result, it is important to monitor the pre-dawn status of athletes to ensure that the regimen adopted does not lead to cumulative deficits in body mass and/or body composition over the month of Ramadan. 

Monitoring these deficits can be increasingly important in adolescent competitors between the ages of 10 and 19 years [16]. These athletes face the added challenges of significant growth and physical development, including alterations of body composition, maturation of organ systems and metabolic and hormonal changes [17]. Nutrients are needed for growth and health maintenance, as well as accommodating the demands of training, competition, school, and work. However, adolescent athletes usually have little nutritional knowledge and are usually reliant on others for the purchase and preparation of foods, which can promote unhealthy eating practices [18,19,20,21,22]. As a result, regular monitoring of nutrition, based on food composition tables to extract accurate dietary information [23] and body composition is important for adolescents, particularly during the observance of Ramadan.

To date, most literature reviews synthesizing the effect of Ramadan observance on body mass and body composition of athletes are narrative in type [24,25,26,27] and require updating. The only systematic reviews and meta-analysis conducted on this topic have focused on sedentary individuals [28,29,30,31]. Moreover, the only systematic review without meta-analysis evaluating the effects of Ramadan observance on dietary intake was conducted in physically active men [32], but not in individuals involved in training and competitive sports.

In the light of this, a systematic review and meta-analysis was conducted to evaluate the effects of Ramadan observance on body mass, body composition and dietary intake in adolescent athletes. It is expected that such a critical examination of existing research will highlight the need for more rigorous future investigations, better controlling potential confounding factors.

## 2. Materials and Methods 

### 2.1. Protocol 

This systematic review followed the Preferred Reporting Items for Systematic reviews and Meta-Analysis (PRISMA) guidelines [33]. 

### 2.2. Eligibility Criteria

Original articles written in English and French languages, and published (or accepted for publication) in peer-reviewed journals were considered. No restrictions were applied in terms of study design, setting, country or time frame. All included articles investigated athletes who continued to train during Ramadan observance. Participants included in the current review were aged between 10 and 19 years [16], and observed the whole month of Ramadan. Descriptive or review articles, conference proceedings, and articles based on either sedentary or obese individuals were excluded. Both single-group pre-post and crossover design studies were accepted, with all of these comparing findings before and during Ramadan observance. Assessment of dietary intake (i.e., total energy intakes, fat intakes, protein intakes, carbohydrates intakes, total water intake) made by a nutritionist and/or determinations of body mass and/or body composition (i.e., absolute body fat, body fat percentage, absolute lean mass) through various techniques were included. Only studies that measured body mass in the early morning were included.

### 2.3. Information Sources and Search

Two electronic databases, PubMed and Web of Science, were searched without applying any time limits or filters; the final search being completed on 12 February 2020. The following combination of keywords was used: [(Ramadan) OR (Ramadan fasting) OR (Ramadan observance)] AND [(adolescent) OR (youth) OR (young) OR (teenager)] AND [(athletes) OR (amateur) OR (professional) OR (player)] AND [(body mass) OR (body weight) OR (body composition) OR (body fat) OR (lean mass) OR (fat free mass) OR (energy intake) OR (nutritional status) OR (caloric intake) OR (dietary intake) OR (water intake)].

In addition, the reference lists of included manuscripts were checked, as well as related citations from other journals identified via Google Scholar and a search of personal files. Specialists in the field were also contacted for information on possible upcoming studies. In addition, specific target journals (Journal of Sports Sciences, Biological Rhythm Research, British Journal of Sports Medicine, Chronobiology International, Journal of Nutrition Fasting and Health, Nutrients, Applied Physiology Nutrition and Metabolism, International Journal of Sport Nutrition and Exercise Metabolism, Journal of the International Society of Sport Nutrition, Science & Sports) were hand-searched for possible accepted studies in the field. 

### 2.4. Study Selection

**Figure 1:**
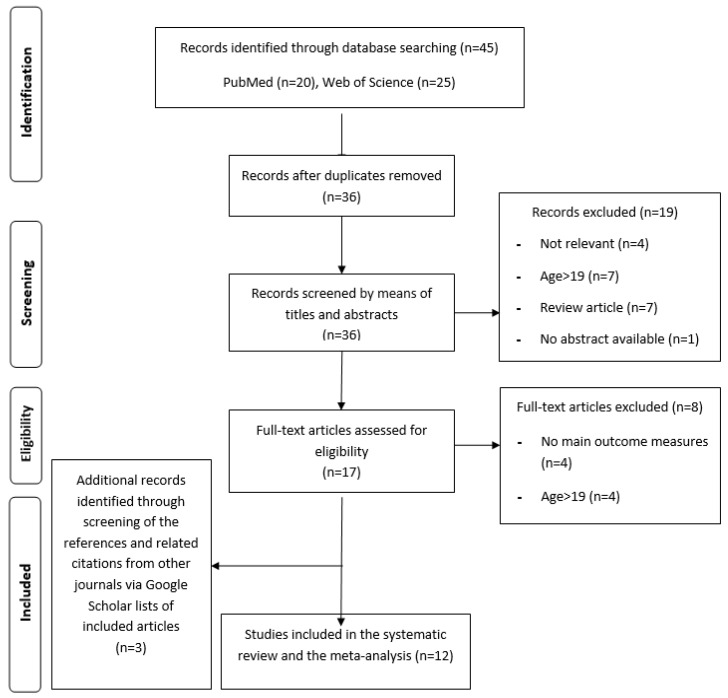
Preferred Reporting Items for Systematic reviews and Meta-Analysis (PRISMA) flow diagram.

The process used for selecting articles is outlined in Figure 1; duplicate articles were eliminated from the initial search using EndNote X8. Two authors independently screened the titles and abstracts of all unique hits for eligibility and resolved disagreements by consensus. The full texts of the selected studies were then screened for eligibility and disagreements were again resolved by consensus; reasons for excluding an article during the full-text screening were recorded. 

### 2.5. Data Collection Process

Two bilingual reviewers (K.T, H.C) independently collected data using a pilot-tested extraction form, and they resolved any disagreements by consensus. Extracted data included participant characteristics (i.e., number of participants, age, sex, sport practiced, type of training program, level of competition and supervision), study characteristics (i.e., country, study design, year of experimental protocol), and key outcomes.

### 2.6. Quality Assessment

The methodological quality of each study was assessed using the formal quantitative assessment tool ‘QualSyst’ [34]. The ‘QualSyst’ rating comprises 14 items (see Tables S1 and S2) that are scored depending on the degree to which a specific criterion is met (yes = 2, partial = 1, no = 0). Items not applicable to a particular study design were marked ‘NA’. A summary score was calculated for each article, adding the total score across relevant items and dividing it by the total possible score. Two reviewers independently performed quality assessments, and disagreements were solved by consensus or by a third reviewer as required. A score of ≥ 75% was considered as indicative of strong quality, a score of 55–75% as moderate quality, and a score of ≤ 55% as weak quality. Additionally, the percentage of lost points within each item was calculated.

### 2.7. Meta-Analysis

Meta-analysis was performed using commercial software (CMA version 3.0, Biostat, Englewood, NJ, USA). Effect sizes (ES) with 95% confidence intervals (CI) were computed according to Cohen. A negative ES value indicated that Ramadan fasting decreased outcomes, while a positive ES indicated that Ramadan fasting increased outcomes. Scores were rated as trivial (ES < 0.2), small (ES 0.2–0.6), moderate (ES 0.6–1.2), large (ES 1.2–2.0), very large (ES > 2.0), or extremely large (ES > 4.0). Q [35] and *I*^2^ [36] statistics were used to assess statistical heterogeneity. An *I*^2^ value > 50% was regarded as evidence of substantial heterogeneity and a random-effect model was then preferred to a fixed-effect model [36]. *I*^2^ value of 25%, 50%, and 75% represent low, moderate, and high statistical heterogeneity, respectively [34]. Given that pre-post correlations were not available in all articles, we followed the recommendations of Rosenthal [37], assuming a conservative estimation of r = 0.7.

Further stratification, relative to the main characteristics, was conducted to identify potential sources of variance and heterogeneity; meta-regression analyses examined quantitative relationships between dependent variables and covariates. Such moderators included population size, age, country, journal impact factor, year of experimental protocol, study quality, type of sport, level of competition, maintenance of training program during Ramadan, number of training sessions per week, duration of training sessions, number of weeks of observation, methods of measuring dietary intake, and the duration of daytime fasting.

Sensitivity analyses assessed the stability of the pooled ES by computing the impact of excluding individual studies from the analysis. A cumulative meta-analysis was also carried out to confirm the stability and reliability of the findings. Funnel plots investigated possible publication bias, looking for potential asymmetries on visual inspection, and carrying out Begg and Mazumdar’s rank correlation test (Kendall’s S statistic P-Q) [38], Egger’s linear regression test [39] and Duval and Tweedie’s trim-and-fill test [40]. A significance level of *p* < 0.05 was adopted for all analyses.

## 3. Results

### 3.1. Study Selection

Our initial search yielded 40 results, of which 17 remained after excluding duplicates and screening titles and abstracts (Figure 1). Nine of the 17 articles met our specific inclusion criteria. A review of reference lists and related citations identified via Google Scholar added 3 additional articles, for a total of 12 reports.

### 3.2. Study Characteristics 

The training characteristics of the included studies is presented in Table S3: body mass data were reported in eight studies [41,42,43,44,45,46,47,48,49,50,51,52] (Table S4) and body composition data were reported in seven studies [41,46,47,48,49,50,51] (Table S5). Güvenç [48] used a foot-to-foot bioelectric impedance analyzer and the remaining studies [41,46,48,49,50,51] used a skinfold caliper to assess body fat content, whilst Meckel et al. [42] reported only the total sum of skinfold thickness; the results of this study were not included in the analysis.

Ten studies assessed dietary intakes during Ramadan [41,42,43,46,47,48,49,50,51,53] using a 2-day diary record [42,48], a 3-day diary record with [41,52] or without interview by a trained nutritionist [43,53] or a 7-day diary record [46,47,49,50] (Table S6). 

Ten articles used a single-group pre-post design without controls [42,45,46,47,48,49,50,51,52,53], two studies used a pretest post-test design with a control group [41,43], and one study used a counterbalanced cross-over design [44].

Eight of the studies were conducted in Tunisia [41,46,47,49,50,51,52,53], two were from Singapore [43,44], and the others were from Turkey [48], Morocco [45] and Israel [42]. 

All participants were men, with mean ages ranging from 13.3 to 18.9 years. A total of 192 athletes were included in the analysis. There were 34 Ramadan observant competitors in the report of Maughan et al. [41] and from 8 to 19 in the remaining studies. Most investigations (*n* = 6) had enrolled soccer players, but studies also included samples of both soccer and basketball players [43], martial arts participants [44,49,50,51,52], and runners [45]. Soccer players enrolled in the studies of Meckel et al. [42] and Güvenç [48] were all playing in the first national division. However, the subjects studied by Maughan et al., [41] included players competing in either a high level (i.e., the first division) or a low level (i.e., the third division). The level of competition in the remaining studies included both amateur [43,44,49,50,51,52,53], and professional athletes [44,46,47].

#### 3.2.1. Effects of Ramadan Fasting on Body Mass

##### Body Mass Before vs. First Week of Ramadan

Six studies, comprising 81 total participants, assessed body mass of adolescent athletes before Ramadan and after one week of Ramadan fasting [43,45,48,49,50,52]. Lotfi et al. [45] reported findings for soccer players and runners, and results for the two disciplines were thus considered as independent studies. Similar findings in terms of mean ± SD of body mass were reported in Zarrouk et al. [51,52], and only one data of Zarrouk et al. [52] was considered for analysis. From before to the first week of Ramadan, body mass did not change in five studies [43,45,48,49,52], decreased in one study [50] and increased in another study [45]. The summarized effects of seven ESs showed a nonsignificant effect (ES = −0.011, standard error or SE = 0.086, 95% CI −0.180 to 0.158, Z-value = −0.126, *p* = 0.899; Figure S1) of Ramadan fasting on body mass. The statistical heterogeneity was low (Q = 1.140, df = 6, *p* = 0.899; *I*^2^ = 0.000%). 

A funnel plot (Figure S2) showed no evidence of publication bias, a conclusion confirmed by Begg and Mazumdar’s rank correlation test (Kendall’s S statistic P-Q = 3.00; tau without continuity correction = 0.143, z = 0.451, *p* = 0.326; tau with continuity correction = 0.095, z = 0.300, *p* = 0.382) and by Egger’s linear regression test (intercept = 0.800, SE = 1.302, 95% CI −2.546 to 4.147, t = 0.615, df = 5, *p* = 0.283). The Duval and Tweedie’s trim-and-fill test did not identify any missing studies.

##### Body Mass Before vs. Second Week of Ramadan

Five studies, comprising 98 participants, assessed body mass of adolescent athletes before Ramadan and after two weeks of Ramadan fasting [41,43,45,46,47]. Maughan et al. [41] reported data of a group tested in the morning and another in the afternoon. Only the results of the morning group were included in the analysis. From before to the second week of Ramadan, body mass did not change in three studies [41,43,45], decreased in two studies [46,47] and increased in one study [45].

The summarized effects of six ESs showed a nonsignificant effect (ES = −0.020, SE = 0.078, 95% CI −0.174 to 0.134, Z-value = −0.258, *p* = 0.796; Figure S3) of two weeks of Ramadan fasting on body mass. The statistical heterogeneity was low (Q = 1.961, df = 5, *p* = 0.855; *I^2^* = 0.00%). 

A funnel plot (Figure S4) showed no evidence of publication bias, a conclusion confirmed by Begg and Mazumdar’s rank correlation test (Kendall’s S statistic P-Q = 1.00; tau without continuity correction = 0.067, z = 0.188, *p* = 0.425; tau with continuity correction = 0.000, z = 0.000, *p* = 0.500) and by Egger’s linear regression test (intercept = 0.341, SE = 1.246, 95% CI −3.218 to 3.800, t = 0.273, df = 4, *p* = 0.399). The Duval and Tweedie’s trim-and-fill test did not identify any missing study.

##### Body Mass Before vs. Fourth Week of Ramadan

Eleven studies, comprising 170 participants, assessed body mass of adolescent athletes before Ramadan and after four weeks of Ramadan fasting [41,42,43,44,45,46,47,48,49,50,52]. In Aziz et al. [44], only body mass results recorded in the morning were analyzed. From before to the fourth week of Ramadan, body mass did not change in eight studies [41,42,43,44,45,48,49,52], decreased in three studies [46,47,50] and increased in one study [45].

**Figure 2:**
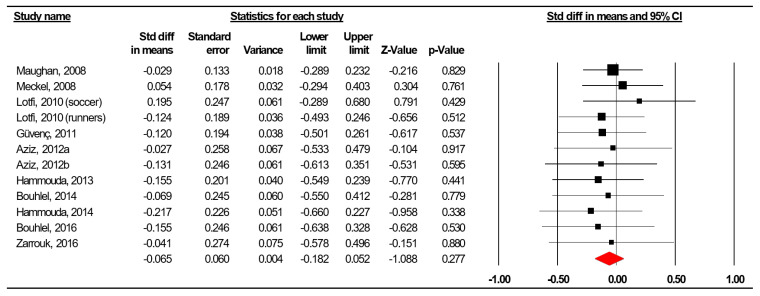
Forest plot for the effect of four weeks of Ramadan observance on body mass in adolescent athletes.

The summarized effects of 12 ESs showed a non-significant effect (ES = −0.069, SE = −0.060, 95% CI −0.182 to 0.052 Z-value = −1.088, *p* = 0.277; Figure 2) of four weeks of Ramadan fasting on body mass. The statistical heterogeneity was low (Q = 2.692, df = 11, *p* = 0.277; *I*^2^ = 0.000%). 

**Figure 3:**
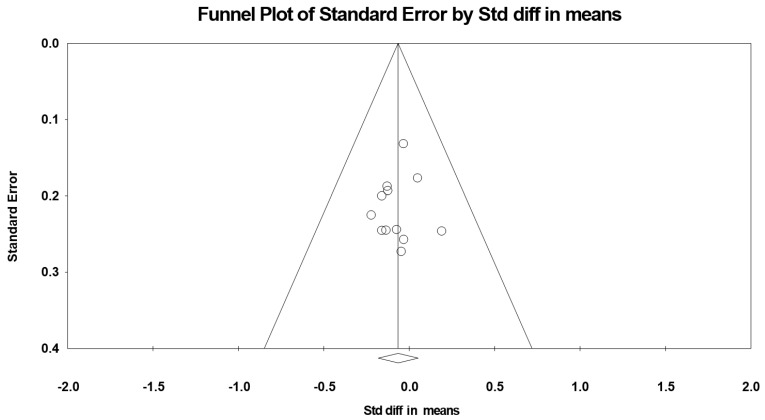
Funnel plot for body mass in adolescent athletes during the fourth week of Ramadan observance, showing no evidence of publication bias.

A funnel plot (Figure 3) showed no evidence of publication bias, a conclusion confirmed by Begg and Mazumdar’s rank correlation test (Kendall’s S statistic P-Q = 4.00; tau without continuity correction = 0.061, z = 0.274, *p* = 0.392; tau with continuity correction = 0.045, z = 0.206, *p* = 0.418) and by Egger’s linear regression test (intercept = −0.250, SE = 0.693, 95% CI −1.795 to 1.295, t = 0.361, df = 10, *p* = 0.363). The Duval and Tweedie’s trim-and-fill test did not identify any missing study.

In conclusion, both sensitivity analysis and cumulative meta-analysis confirmed the reliability and stability of the current findings. 

#### 3.2.2. Effects of Ramadan Fasting on Body Composition

##### Effects of Ramadan Fasting on Body Fat Mass (kg)

Body Fat Before vs. First Week of Ramadan:

Three studies, comprising 28 participants, assessed body fat of adolescent athletes before Ramadan and after one week of Ramadan fasting [49,50,51]. From before to the first week of Ramadan, body fat did not change in these three studies [49,50,51].

The summarized effects of three ESs showed a nonsignificant effect (ES = 0.104, SE = 0.147, 95% CI −0.184 to 0.393, Z-value = 0.707, *p* = 0.480; Figure S5) of one week of Ramadan fasting on body fat. The statistical heterogeneity was low (Q = 0.553, df = 2, *p* = 0.759; *I*^2^ = 0.000%). 

A funnel plot (Figure S6) showed no evidence of publication bias, a conclusion confirmed by Begg and Mazumdar’s rank correlation test (Kendall’s S statistic P-Q =1.00; tau without continuity correction = 0.333, z = 0.522, *p* = 0.301; tau with continuity correction = 0.000, z = 0.000, *p* = 0.500) and by Egger’s linear regression test (intercept = 1.765, SE = 8.302, 95% CI −103.720 to 107.255, t = 0.213, df = 1, *p* = 0.433). The Duval and Tweedie’s trim-and-fill test did not identify any missing study.

Body Fat Before vs. Fourth Week of Ramadan:

Four studies, comprising 40 participants, assessed body fat of adolescent athletes before Ramadan and after four weeks of Ramadan fasting [47,49,50,51]. From before to the fourth week of Ramadan, body fat did not change in three studies [49,50,51] and decreased in one study [47].

The summarized effects of four ESs showed a nonsignificant effect (ES = 0.018, SE = 0.124, 95% CI −0.224 to 0.261, Z-value = 0.141, *p* = 0.882; Figure S7) of Ramadan fasting on body fat. The statistical heterogeneity was low (Q = 2.639, df = 3, *p* = 0.451; *I*^2^ = 0.000%). 

A funnel plot (Figure S8) showed no evidence of publication bias, a conclusion confirmed by Begg and Mazumdar’s rank correlation test (Kendall’s S statistic P-Q = 4.00; tau without continuity correction = 0.667, z =1.359, *p* = 0.087; tau with continuity correction = 0.500, z = 0.019, *p* = 0.154) and by Egger’s linear regression test (intercept = 6.551, SE = 6.831, 95% CI −22.838 to 35.941, t = 0.959, df = 2, *p* = 0.219). The Duval and Tweedie’s trim-and-fill test did not identify any missing study.

In conclusion, both sensitivity analysis and cumulative meta-analysis confirmed the reliability and stability of the current findings. 

##### Effects of Ramadan Fasting on Body Fat Percentage (%)

Body Fat Before vs. Second Week of Ramadan:

Two studies, comprising 49 participants, assessed body fat percentage of adolescent athletes before Ramadan and after two weeks of Ramadan fasting [41,46]. From before to the second week of Ramadan, body fat percentage remained unchanged in the two studies [41,46]. The summarized effects of two ESs showed a nonsignificant effect (ES = 0.013, SE = 0.111, 95% CI −0.204 to 0.230, Z-value = 0.121, *p* = 0.904; Figure S9) of two weeks of Ramadan fasting on body fat percentage. The statistical heterogeneity was low (Q = 0.059, df = 1, *p* = 0.808; *I*^2^ = 0.000%). 

Body Fat Before vs. Fourth Week of Ramadan:

Three studies, comprising 65 participants, assessed body fat percentage of adolescent athletes before Ramadan and after four weeks of Ramadan fasting [41,46,48]. From before to the fourth week of Ramadan, body fat percentage decreased in one study [46] and did not change in two studies [41,48].

The summarized effects of three ESs showed a nonsignificant effect (ES = −0.093, SE = 0.096, 95% CI −0.282 to 0.095, Z-value = −0.969, *p* = 0.333; Figure S10) of one week of Ramadan fasting on body fat. The statistical heterogeneity was low (Q = 0.122, df = 2, *p* = 0.941; *I*^2^ = 0.000%). 

A funnel plot (Figure S11) showed no evidence of publication bias, a conclusion confirmed by Begg and Mazumdar’s rank correlation test (Kendall’s S statistic P-Q = −1.00; tau without continuity correction = −0.333, z = 0.522, *p* = 0.301; tau with continuity correction = 0.000, z = 0.000, *p* = 0.500) and by Egger’s linear regression test (intercept = −0.792, SE = 0.679, 95% CI −9.418 to 7.834, t = 1.166, df = 1, *p* = 0.226). The Duval and Tweedie’s trim-and-fill test did not identify any missing study.

#### 3.2.3. Effects of Ramadan Fasting on Lean Mass (kg)

##### Lean Mass Before vs. Fourth Week of Ramadan

Two studies, comprising 23 participants, assessed lean mass of adolescent athletes before Ramadan and after four weeks of Ramadan fasting [46,51]. From before to the fourth week of Ramadan, lean mass did not change in these two studies [46,51].

The summarized effects of two ESs showed a nonsignificant effect (ES = −0.025, SE = 0.162, 95% CI −0.343 to 0.292, Z-value = −0.156, *p* = 0.876; Figure S12) of Ramadan fasting on lean mass. The statistical heterogeneity was low (Q = 0.483, df = 1, *p* = 0.487; *I*^2^ = 0.000%). 

#### 3.2.4. Effects of Ramadan Fasting on Dietary Intake

##### Total Energy Intake Before vs. During Ramadan

Ten studies, comprising 162 participants, evaluated the effect of Ramadan fasting on total energy intake in adolescent athletes [41,42,43,46,47,48,49,50,52,53]. From before to during Ramadan, total energy intake did not change in four studies [42,43,48,53], increased in two studies [41,52] and decreased in four studies [46,47,49,50].

**Figure 4:**
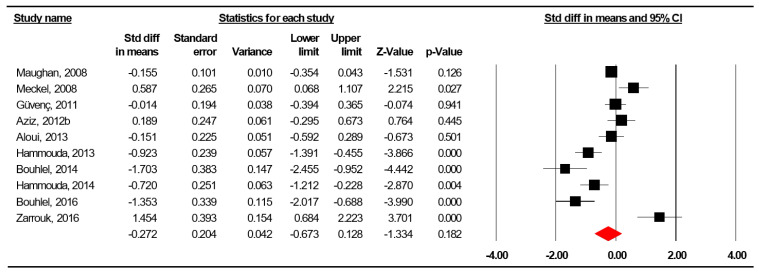
Forest plot for the effect of Ramadan observance on total energy intakes in adolescent athletes.

The summarized effects of ten ESs showed a nonsignificant effect (ES = −0.272, SE = 0.204, 95% CI −0.673 to −0.128, Z-value = −1.334, *p* = 0.182; Figure 4) of Ramadan fasting on total energy intake. The statistical heterogeneity was high (Q = 70.546, df = 9, *p* < 0.0001; *I^2^* = 87.242%).

**Figure 5:**
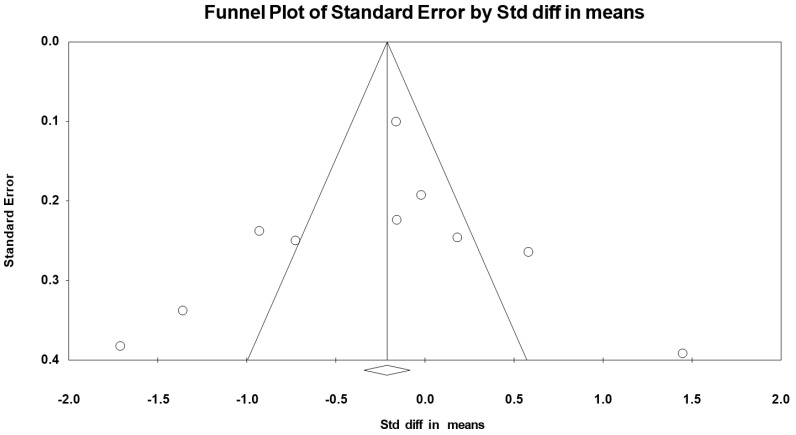
Funnel plot for total energy intakes in adolescent athletes during Ramadan observance showing no evidence of publication bias.

A funnel plot (Figure 5) showed no evidence of publication bias, a conclusion confirmed by Begg and Mazumdar’s rank correlation test (Kendall’s S statistic P-Q = −3.00; tau without continuity correction = −0.667, z = 0.268, *p* = 0.394; tau with continuity correction = −0.044, z = 0.179, *p* = 0.429) and by Egger’s linear regression test (intercept = −0.892, SE = 2.232, 95% CI −6.04 to 4.255, t = 0.4, df = 8, *p* = 0.40). The Duval and Tweedie’s trim-and-fill test did not identify any missing study.

Among the meta-regressions, no impact of sample size (coefficient = 0.0005, SE = 0.0163, (95% CI −0.0315 to 0.0325), z = 0.03, *p* = 0.975), age (coefficient = −0.211, SE = 0.132, (95% CI −0.470 to 0.048), z = −1.6, *p* = 0.110), country of study (Q = 2.77, degrees of freedom = 3, *p* = 0.428), journal impact factor (coefficient = 0.397, SE = 0.190, (95% CI 0.024 to 0.770), z = 2.08, *p* = 0.037), year of measurement (coefficient = −0.225, SE = 0.229, (95% CI −0.675 to 0.770), z = −0.98, *p* = 0.035), study quality score (coefficient = 0.036, SE = 0.022, (95% CI −0.008 to 0.008), z = 1.62, *p* = 0.105), type of physical activity (Q = 4.51, degrees of freedom = 4, *p* = 0.341), level of competition (Q = 2.37, degrees of freedom = 3, *p* = 0.499), degree of maintenance of training program (Q = 0.93, degrees of freedom = 2, *p* = 0.629), number of training sessions per week (coefficient = −0.048, SE = 0.633, (95% CI −1.528 to 0.958), z = −0.45, *p* = 0.656), average duration of training sessions (coefficient = −0.282, SE = 0.239, (95% CI −0.517 to 0.420), z = −0.2, *p* = 0.839), or the number of weeks of measurement (coefficient = 0.304, SE = 0.216, (95% CI −0.120 to 0.728), z = 1.41, *p* = 0.160) could be identified. Interestingly, the meta-regression found a statistically significant value when stratified on the method of dietary intakes measurement (Q = 15.25, degrees of freedom = 3, *p* = 0.002) as well as on daytime duration of fasting (coefficient = −0.773, SE = 0.258, (95% CI −1.280 to −0.267), z = −2.99, *p* = 0.003). 

##### Protein Intake (g) Before vs. During Ramadan

Five studies, comprising 114 participants, evaluated the effect of Ramadan fasting on protein intake in adolescent athletes [41,46,47,50,52]. As compared to before Ramadan, protein consumption during Ramadan remained unchanged in four studies [46,47,52], increased in one study [41] and decreased in another additional study [50].

The summarized effects of five ESs showed a non-significant effect (ES = 0.069, SE = 0.192, 95% CI −0.308 to 0.446, Z-value = 0.359, *p* = 0.720; Figure S13) of Ramadan fasting on protein intake. The statistical heterogeneity was high (Q = 17.576, df = 4, *p* = 0.001; *I*^2^ = 77.473%). 

A funnel plot (Figure S14) showed no evidence of publication bias, a conclusion confirmed by Begg and Mazumdar’s rank correlation test (Kendall’s S statistic P-Q = −2.00; tau without continuity correction = −0.2, z = 0.490, *p* = 0.312; tau with continuity correction = −0.1, z = 0.245, *p* = 0.312) and by Egger’s linear regression test (intercept = −2.998, SE = 2.144, 95% CI −9.820 to 3.823, t = 1.399, df = 3, *p* = 0.128). The Duval and Tweedie’s trim-and-fill test did not identify any missing study.

##### Fat Intake (g) Before vs. During Ramadan

Five studies, comprising 104 participants, evaluated the effect of Ramadan fasting on fat intake in adolescent athletes [41,46,47,50,52]. From before to during Ramadan, fat intake decreased in three studies [46,47,50], remained unchanged in one [41] and increased in another study [52].

The summarized effects of five ESs showed a nonsignificant effect (ES = 0.044, SE = 0.221, 95% CI −0.390 to 0.478, Z-value = 0.199, *p* = 0.842; Figure S15) of Ramadan fasting on total fat intake. The statistical heterogeneity was high (Q = 21.793, df = 4, *p* = 0.000; *I*^2^ = 81.646%).

A funnel plot (Figure S16) showed no evidence of publication bias, a conclusion confirmed by Begg and Mazumdar’s rank correlation test (Kendall’s S statistic P-Q = 0.000; tau without continuity correction = 0.000, z = 0.000, *p* = 0.500; tau with continuity correction = 0.000, z = 0.000 *p* = 0.500) and by Egger’s linear regression test (intercept = 2.260, SE = 1.759, 95% CI −3.538 to 7.658, t = 1.171, df = 3, *p* = 0.163). The Duval and Tweedie’s trim-and-fill test did not identify any missing studies.

##### Carbohydrates Intake (g) Before vs. During Ramadan

Five studies, comprising 104 participants, evaluated the effect of Ramadan fasting on carbohydrates intake in adolescent athletes [41,46,47,50,52]. From before to during Ramadan, carbohydrates intake remained unchanged in four studies [41,46,47,52] and increased in one [50].

The summarized effects of five ESs showed a nonsignificant effect (trivial ES = 0.075, SE = 0.146, 95% CI −0.211 to 0.362, Z-value = 0.516, *p* = 0.606; Figure S17) of Ramadan fasting on carbohydrates intake. The statistical heterogeneity was moderate (Q = 10.574, df = 4, *p* = 0.032; *I*^2^ = 62.170%).

A funnel plot (Figure S18) showed no evidence of publication bias, a conclusion confirmed by Begg and Mazumdar’s rank correlation test (Kendall’s S statistic P-Q=0.000; tau without continuity correction = 0.000, z = 0.000, *p* = 0.500; tau with continuity correction=0.000, z = 0.000, *p* = 0.500) and by Egger’s linear regression test (intercept = 0.699, SE = 2.663, 95% CI -7.775 to 9.173, t = 1.262, df = 3, *p* = 0.405). The Duval and Tweedie’s trim-and-fill test did not identify any missing studies.

##### Total Water Intake (L) Before vs. During Ramadan

Five studies, comprising 97 participants, evaluated the effect of Ramadan fasting on total water intake in adolescent athletes [41,43,49,50,52]. From before to during Ramadan, total water intake remained unchanged in two studies [41,43], decreased in two studies [49,50] and increased in one study [52].

The summarized effects of five ESs showed a nonsignificant effect (ES = −0.115, SE = 0.235, 95% CI −0.576 to 0.346, Z-value = −0.490, *p* = 0.624; Figure S19) of Ramadan fasting on total water intake. The statistical heterogeneity was high (Q = 21.685, df = 4, *p* = 0.000; *I*^2^ = 81.554%). 

A funnel plot (Figure S20) showed no evidence of publication bias, a conclusion confirmed by Begg and Mazumdar’s rank correlation test (Kendall’s S statistic P-Q = 0.000; tau without continuity correction = 0.000, z = 0.000, *p* = 0.500; tau with continuity correction = 0.000, z = 0.000, *p* = 0.500) and by Egger’s linear regression test (intercept = 0.857, SE=2.599, 95% CI −7.414 to 9.128, t = 0.330, df = 3, *p* = 0.382). The Duval and Tweedie’s trim-and-fill test did not identify any missing studies.

In conclusion, both sensitivity analysis and cumulative meta-analysis confirmed the reliability and stability of the current findings. 

### 3.3. Quality Assessment

Of the twelve selected articles evaluating body mass and/or body composition, one was of strong quality and the remaining studies were rated as moderate. The largest number of points were lost due to inappropriate study design (87.5%) and a weak allowance for confounding factors (95.8%) (Table S1). Ten articles evaluated dietary intake; six were rated as moderate and the remaining four were rated as strong. Causes of lost points included an inappropriate study design (90%), a poor control of confounding factors (50%), and an inappropriate selection of subjects (45%) (Table S2).

## 4. Discussion

This is the first meta-analysis evaluating the effects of Ramadan observance on body mass, body composition and dietary intake in adolescent athletes. A secondary goal of this article looked critically at shortcomings in the published literature. The studies reviewed in this meta-analysis support the conclusion that continuance of training (at least three sessions/week) during the month of Ramadan observance does not change body mass, body composition and dietary intake of adolescent athletes.

During the religious month of Ramadan, adolescent Muslim athletes continue to train and compete, while facing significant changes in eating habits (e.g., number of meals, meals content, exclusive nocturnal meals), sleeping, activity patterns, and circadian rhythms of hormones such as cortisol, insulin, leptin, adiponectin, ghrelin, prolactin growth hormone, and sex hormones; these changes may also be accompanied by subsequent changes in energy metabolism [54]. In a previous investigation, Coyle et al. [55] demonstrated that fatty acid oxidation increased, independent of decreases in carbohydrate oxidation, even after a few hours of fasting. Therefore, it is not surprising that many investigators have evaluated the possible cumulative changes that would occur in body mass and body composition during the month of Ramadan. The present results indicate that one week or two weeks of Ramadan observance does not affect body mass and body composition of adolescent athletes. It is possibly that the short duration of intermittent fasting was not sufficient to exert significant effects on body composition. Similarly, compared to before Ramadan, body mass, body fat, and lean tissue mass did not change compared to the end of the Ramadan month. This suggests that adolescent Muslim athletes compensate for the energy deficits incurred during the daylight hours of Ramadan. Furthermore, the current meta-analysis demonstrated that total energy intakes remained unchanged during compared to before Ramadan observance. Another possible explanation of the unchanged body mass during Ramadan is the absence of change in total water intake, arguing against any cumulative hypohydration. This is important as a significant level of dehydration can result in impairments to physical and cognitive performance [56]. To the best of the authors’ knowledge, no studies conducted in either adolescent or adult athletes report any significant decreases in lean mass, suggesting that training can continue during Ramadan without the adverse consequence of muscle protein metabolism.

Our meta-analysis results on body mass and body composition are not without limitations. First, despite the fact that the inherent physical stress of training sessions and competition during a season can modulate body composition [57], the volume and intensity of training sessions during Ramadan were described only by Maughan et al. [41] and Meckel et al. [42]. The training load in the study of Maughan et al. [41] was not markedly changed (rating of perceived effort = 13 (6–20)) before compared to during Ramadan, or between the fasting and non-fasting groups. Additionally, the number, intensity, and duration of training sessions were maintained during compared to before Ramadan. In this study, players participated in 6–8 training sessions of 1.5 h per week [41]. Meckel et al. [42] reported a significant reduction in intense weekly physical exercise from 6.4 h before Ramadan to 4.5 h during Ramadan. In this study, coaches modified training programs by increasing low intensity, technical, coordination/tactical training, and by reducing high intensity training. In the study of Meckel et al. [42], the same training volume was maintained during as before Ramadan: three 1.5 h training sessions a week performed at 5 p.m. and a match during the weekend. In the remaining studies, the authors claimed that training characteristics were maintained during compared to before Ramadan; however, no objective measurement (e.g., heart rate, global positioning system) has been performed to confirm their claims. The poor documentation of the training plans needs to be addressed in future research.

Second, there are issues related to the methods utilized for the assessment of body mass and body composition. Regarding the body fat content assessment, Güvenç [48] used the bioelectric impedance; however, body fat content has been estimated from skinfold thicknesses in the remaining studies [49,50,51,52]. It is noteworthy that adipose tissue contains 20% water [58], and the compressibility of skinfolds may be influenced by dehydration. Our data did not point to significant cumulative dehydration, but other reports have found athletes to become hypohydrated [59,60], limiting the reliability of skinfold estimates on changes in body fat content. Several studies also failed to discuss the technical abilities of the anthropometrist; Stewart et al. [61] recommended that anthropometric measurements should always follow the protocol of the International Society for the Advancement of Kinanthropometry. Unfortunately, none of the studies included in this meta-analysis provided details of the protocol adopted. For more accurate measurement of body fat, the use of a “gold standard” noninvasive method (e.g., DEXA) in future studies of Ramadan is warranted.

Regarding body mass measurement, it is recommended that subjects should void and urinate in the early morning, before the measurement of body mass [62]. However, this precaution was reported only by Aziz et al. [43] and may not have been observed in the other studies to date. Clearly, future studies during Ramadan should take more precautions during the measurement of body mass.

Our meta-analysis findings on the effects of Ramadan observance on body mass of adolescent athletes are not in line with those conducted on sedentary subjects. Kul et al. [28] and Sadeghirad et al. [29] concluded that fasting during Ramadan resulted in significant body mass reduction. The common explanations of this decrease in body mass during Ramadan are the restrictions in meal frequencies or energy intake, coupled with the combination of different factors such as the efficient utilization of body fat stores during Ramadan, dehydration, physical activity level, the lower absorption of foods eaten nocturnally during Ramadan, and sleeping patterns and duration [28,29]. Similar findings have been reported by Fernando et al. [30]; but these findings have been criticized because the authors included studies on physical activity during Ramadan observance in their analysis, which could limit the level of evidence of the meta-analysis.

To overcome this shortcoming reported by Fernando et al. [30], Jahrami et al. [31] reported a significant, but small, reduction in body mass during Ramadan month in sedentary individuals. Similar explanations as those of Kul et al. [28], Sadeghirad et al. [29] and Fernando et al. [30] have been proposed to explain the decrease in body mass during Ramadan in sedentary subjects.

The contradictory findings of our meta-analysis and those conducted on sedentary individuals [27,28,29,30,31] could be explained by differences in the quantity and quality of food intake, water intake, age, gender and energy expenditure. Moreover, some sedentary individuals use Ramadan in an attempt to reduce body fat content [25].

Our results have also demonstrated heterogeneous findings regarding the effects of Ramadan observance on total energy intakes from before compared to during Ramadan. The current meta-regression indicates that the estimated energy intake is influenced by the duration of daytime fasting and the method of dietary intake assessment. An under-reporting of habitual energy intake is common among athletes [63] and duration of data collection is a controversial issue. Four studies [46,47,49,50] have used 7-day records, arguing that there are important differences in food consumption between weekdays and weekend days. However, if recording continues for more than 4 consecutive days, a decrease in the reported intakes may likely be observed due to respondent fatigue [64,65]. It appears that habitual energy intakes recorded in the previous studies [46,47,49,50] were under-reported. The other studies evaluating the effects of Ramadan observance on energy intakes used a 2-day record without interview [42,48] or a 3-day record with [41,52] or without interview [43,53]. It has been reported that respondents with interviewer probing reported 25% higher dietary intakes than did respondents without interviewer probing [66]. Taken together, the results of habitual energy intakes in almost all studies [42,43,46,47,48,49,50,53] could be under-reported. This issue needs to be addressed in future research studies by coupling the collection of nutritional information for four nonconsecutive days with the interview of the participants by a dietician [65].

In the studies included in the present meta-analysis, athletes had differing levels of competition. For example, in the studies of Maughan et al. [41] and Hammouda et al. [46,47], soccer players were professional, living in an athlete’s village, with diet and mealtimes determined by the coach to fit times of training and competition; consequently muscle mass and the athlete’s hydration status could be preserved. However, in the remaining reports including amateur athletes [49,50,51,52], it is unclear whether subjects received advice about the amount and the quality of food to consume or faced other constraints in obtaining the type and amount of food that they wanted. 

## 5. Strengths and Weaknesses

The strengths of the present study include the comprehensive coverage of the available literature and the careful appraisal of its quality. However, the number of good quality studies evaluating changes in body mass and body composition are limited, hampering the ability to draw definitive conclusions. Other significant issues need to be addressed in future studies. In almost all studies conducted to date, the sample size has been small, and the minimum number of subjects has not been calculated [67]. Additionally, with the exception of the studies by Maughan et al. [41] and Aziz et al. [43], control groups have been lacking, to date. Recruitment of matched control subjects is difficult in Muslim countries; but controls are vital to detect effects from seasonal changes. Such controls must be recruited from the same group of athletes, sharing the same living quarters, and facing the same practical constraints, such as altered mealtimes.

## 6. Conclusions

Available data suggests that the continuation of training at least three times/week during Ramadan observance has no effect on body mass, body composition, or dietary intake in adolescent athletes. Future studies with greater methodological rigor, particularly around dietary intake and body composition, are warranted.

## Supplementary Materials

The following are available online at https://www.mdpi.com/2072-6643/12/6/1574/s1, Figure S1: Forest plot for the effect of one week of Ramadan observance on body mass in adolescent athletes, Figure S2: Funnel plot of body mass for adolescent athletes during the first week of Ramadan observance showing no evidence of publication bias, Figure S3: Forest plot for the effect of two weeks of Ramadan observance on body mass in adolescent athletes, Figure S4: Funnel plot for body mass in adolescent athletes during the second week of Ramadan observance, showing no evidence of publication bias, Figure S5: Forest plot for the effect of one week of Ramadan observance on body fat mass in adolescent athletes, Figure S6: Funnel plot for body fat mass in adolescent athletes during the first week of Ramadan observance, showing no evidence of publication bias, Figure S7: Forest plot for the effect of four weeks of Ramadan observance on body fat mass in adolescent athletes, Figure S8: Funnel plot for body fat mass in adolescent athletes during the fourth week of Ramadan observance, showing no evidence of publication bias, Figure S9: Forest plot for the effect of two weeks of Ramadan observance on body fat percentage in adolescent athletes, Figure S10: Forest plot for the effect of four weeks of Ramadan observance on body fat percentage in adolescent athletes, Figure S11: Funnel plot for the effect of four weeks of Ramadan observance on body fat percentage in adolescent athletes, showing no evidence of publication bias, Figure S12: Forest plot for the effect of four weeks of Ramadan observance on lean mass in adolescent athletes, Figure S13: Forest plot for the effect of Ramadan observance on protein intake in adolescent athletes, Figure S14: Funnel plot for protein intake in adolescent athletes during Ramadan observance showing no evidence of publication bias, Figure S15: Forest plot for the effect of Ramadan observance on fat intakes in adolescent athletes, Figure S16: Funnel plot for fat intakes in adolescent athletes during Ramadan observance showing no evidence of publication bias, Figure S17: Forest plot for the effect of Ramadan observance on carbohydrates intakes in adolescent athletes, Figure S18: Funnel plot for carbohydrates intakes in adolescent athletes during Ramadan observance showing no evidence of publication bias, Figure S19: Forest plot for the effect of Ramadan observance on total water intakes in adolescent athletes, Figure S20: Funnel plot for total water intakes in adolescent athletes during Ramadan observance showing no evidence of publication bias, Table S1: Quality assessment of body composition, Table S2: Quality assessment of dietary intake, Table S3: Training characteristics of the included studies, Table S4: Effect of Ramadan fasting on body mass, Table S5: Effect of Ramadan fasting on body composition, Table S6: Effect of Ramadan fasting on dietary intake.
