# Supplementary material for: Effects of Ramadan Observance on Dietary Intake and Body Composition of Adolescent Athletes: Systematic Review and Meta-Analysis
Source: Nutrients. 2020 May 28;12(6):1574. doi: 10.3390/nu12061574 (PMC7353054; doi:10.3390/nu12061574)
Supplement: Supplementary file 1 [file nutrients-12-01574-s001.pdf]

Supplementary files

Table S1. Quality assessment of body composition.

Study	Question described	Appropriate study design	Appropriate subject selection	Characteristics described	Random allocation	Researchers blinded	Subjects blinded	Outcome measures well defined and robust to bias	Sample size appropriate	Analytic methods well described	Estimate of variance reported	Controlled for confounding	Results reported in detail	Conclusion supported by results?	Rating	Study quality
**Maughan et al. [41]**	2	1	2	2	n/a	n/a	n/a	2	2	2	2	1	2	2	90.9	strong
**Meckel et al. [42]**	2	0	1	1	n/a	n/a	n/a	1	1	1	2	0	2	2	59.1	moderate
**Lotfi et al. [45]**	2	0	1	1	n/a	n/a	n/a	1	2	1	2	0	2	2	63.6	moderate
**Güvenç [48]**	2	0	1	2	n/a	n/a	n/a	1	1	1	2	0	2	2	63.6	moderate
**Aziz et al. [43]**	2	1	1	2	n/a	n/a	n/a	1	1	1	2	0	2	2	68.2	moderate
**Aziz et al. [44]**	2	1	1	2	n/a	n/a	n/a	1	1	1	2	0	2	2	68.2	moderate
**Aloui et al. [53]**	2	0	1	1	n/a	n/a	n/a	1	1	1	2	0	2	2	59.1	moderate
**Hammouda et al. [46]**	2	0	1	2	n/a	n/a	n/a	1	1	1	2	0	2	2	63.6	moderate
**Bouhlel et al. [49]**	2	0	1	1	n/a	n/a	n/a	1	1	1	2	0	2	2	59.1	moderate
**Hammouda et al. [47]**	2	0	1	2	n/a	n/a	n/a	1	1	1	2	0	2	2	63.6	moderate
**Bouhlel et al. [50]**	2	0	1	2	n/a	n/a	n/a	1	1	1	2	0	2	2	63.6	moderate
**Zarrouk et al. [51]**	2	0	1	2	n/a	n/a	n/a	1	1	1	2	0	2	2	63.6	moderate
% of lost points	0	87.5	45.8	16.6	n/a	n/a	n/a	45.8	41.6	45.8	0	95.8	0	0		

n/a: not applicable.

Table S2. Quality assessment of dietary intake.

Study	Question described	Appropriate study design	Appropriate subject selection	Characteristics described	Random allocation	Researchers blinded	Subjects blinded	Outcome measures well defined and robust to bias	Sample size appropriate	Analytic methods well described	Estimate of variance reported	Controlled for confounding	Results reported in detail	Conclusion supported by results?	Rating	Study quality
**Maughan et al. [41]**	**2**	**1**	**2**	**2**	**n/a**	**n/a**	**n/a**	**2**	**2**	**2**	**2**	**1**	**2**	**2**	**90.9**	Strong
**Meckel et al. [42]**	2	0	1	1	n/a	n/a	n/a	2	1	1	2	1	1	1	59.09	Moderate
**Güvenç. [48]**	2	0	1	2	n/a	n/a	n/a	2	2	1	2	1	2	2	77.27	Strong
**Aziz et al. [43]**	2	1	1	2	n/a	n/a	n/a	1	1	2	2	1	2	2	77.27	Strong
**Aloui et al. [53]**	2	0	1	1	n/a	n/a	n/a	2	1	1	2	1	2	2	68.18	Moderate
**Hammouda et al. [46]**	2	0	1	2	n/a	n/a	n/a	0	1	1	1	1	2	2	59.09	Moderate
**Bouhlel et al. [49]**	2	0	1	1	n/a	n/a	n/a	0	1	1	2	1	2	2	59.09	Moderate
**Hammouda et al. [47]**	2	0	1	2	n/a	n/a	n/a	1	1	1	2	1	2	2	68.18	Moderate
**Bouhlel et al. [50]**	2	0	1	2	n/a	n/a	n/a	0	1	1	2	1	2	2	63.63	Moderate
**Zarrouk et al. [52]**	2	0	1	2	n/a	n/a	n/a	2	1	2	2	1	2	2	77.27	Strong
% of lost points	0	90	45	15	n/a	n/a	n/a	40	40	35	5	50	5	5	-	-

n/a: not applicable.

Table S3. Training characteristics of the included studies.

Studies	Activity	Level of practice	Time of day of training during Ramadan	Number of training sessions	Duration of training sessions (h)	Timing of measurement	Training program
**Maughan et al. [41]**	Soccer	First and third division	In the afternoon before breaking the fast	7	1	In the morning<br>In the afternoon	Training program maintained during compared to before Ramadan
**Meckel et al. [42]**	Soccer	Amateur	-	-	-	-	High intensities activities decreased during compared to before Ramadan
**Lotfi et al. [45]**	Soccer<br>Running	Professional	-	3	-	-	-
**Güvenç [48]**	Soccer	Amateur	After breaking the fast	3	2	In the afternoon	Training program maintained during compared to before Ramadan
**Aziz et al. [43]**	Soccer or basketball	Amateur	Before breaking the fast	3	1	-	
**Aziz et al. [44]**	Martial arts sport of pencaksilat	Amateur	In the late afternoon or evening	3-4	1-1.5	08h00<br>18h00<br>21h00	
**Hammouda et al. [46]**	Soccer	Professional	After breaking the fast	4	2	In the morning	
**Zarrouk et al. [51]**	Karate	Amateur	After breaking the fast	5	2	In the afternoon	
**Bouhlel et al. [49]**	Karate	Amateur	-	5	2	-	
**Hammouda et al. [47]**	Karate	Amateur	-	5	2	-	
**Bouhlel et al. [50]**	Boxing	Amateur	-	5	1.5	In the afternoon	
**Zarrouk et al. [52]**	Karate	Amateur	After breaking the fast	5	2	-	

Table S4. Effect of Ramadan fasting on body mass.

Studies	Sample size	Age	Country	Year of experimental protocol	Fasting hours	Study design	Measured parameter	Effect
**Maughan et al. [41]**	34 (a.m)	18.5	Tunisia	2006	14.5	Pre/post-control	Body mass (kg)	↔
	25 (p.m)							↔
**Meckel et al. [42]**	19	15.1	Israel	-	-	Pre/post	Body mass (kg)	↔
**Lotfi et al. [45]**	10 (soccer)	15.8	Morocco	-	-	Pre/post	Body mass (kg)	↔
	17 (runners)	16.8						↑ 2.85% at R1 and ↑ 2.74% at R4 vs. Bef-R
**Güvenç [48]**	16	17.4	Turkey	-	-	Pre/post	Body mass (kg)	↔
**Aziz et al. [43]**	10	17.5	Singapore	-	13.5	Pre/post-control	Body mass (kg)	↔
**Aziz et al. [44]**	9	18.9	Singapore	-	13.7	Counterbalanced cross-over design	Body mass (kg)	↔
								↓ 2.08% at Ramadan vs. Bef-R
								↔
**Hammouda et al. [46]**	15	17.3	Tunisia	2010	15.5	Pre/post	Body mass (kg)	↓ 1.96% at R2 and ↓ 3.60% at R4 vs. Bef-R
**Zarrouk et al. [51]**	8	17.2	Tunisia	2009	14	Pre/post	Body mass (kg)	↔
**Bouhlel et al. [49]**	10	18.5	Tunisia	-	-	Pre/post	Body mass (kg)	↔
**Hammouda et al. [34]**	10	18.5	Tunisia	-	-	Pre/post	Body mass (kg)	↓ 2.81 at R2. and ↓ 4.14% at R4 vs. Bef-R
**Bouhlel et al. [50]**	10	18.8	Tunisia	2011	15	Pre/post	Body mass (kg)	↓ 1% at R4 vs. Bef-R
**Zarrouk et al. [52]**	8	17.2	Tunisia	2009	14	*Pre/post	Body mass (kg)	↔

NM = not mentioned; ↓ = decrease; ↔ = no-change; ↑ = increase; *vs.*= versus; Bef-R = Before Ramadan; R1 = First week of Ramadan; R2 = Second week of Ramadan; R4 = Fourth week of Ramadan.

Table S5. Effect of Ramadan fasting on body composition.

Studies	Sample size	Age	Country	Year of experimental protocol	Fasting hours	Study design	Measured parameters	Effect
**Maughan et al. [41]**	34 (a.m)	18.5	Tunisia	2006	14.5	Pre/post-control	Body fat (%)	↔
	25 (p.m)							↔
**Güvenç [48]**	16	17.4	Turkey			Pre/post	Body fat (%)	↔
							Fat-free mass (Kg)	↔
**Hammouda et al. [46]**	15	17.3	Tunisia	2010	15.5	Pre/post	Fat mass (%)	↓ 3.7% at R4 vs. Bef-R
							Lean mass (kg)	↔
**Zarrouk et al. [51]**	8	17.2	Tunisia	2009	14	Pre/post	Fat mass (kg)	↔
							Lean mass (kg)	↔
**Bouhlel et al. [49]**	10	18.5	Tunisia			Pre/post	Fat mass (kg)	↔
**Hammouda et al. [47]**	10	18.5	Tunisia			Pre/post	Fat mass (kg)	↓ 6.71% at R4 vs. Bef-R
**Bouhlel et al. [50]**	10	18.8	Tunisia	2011	15	Pre/post	Body fat (%)	↔
							Fat mass (kg)	↔
							Fat- free mass (kg)	↓ 2.71% at R4 vs. Bef-R

NM = not mentioned; ↓ = decrease; ↔ = no-change; ↑ = increase; *vs.*= versus; Bef-R = Before Ramadan; R4 = Fourth week of Ramadan.

Table S6. Effect of Ramadan fasting on dietary intake.

Studies	Sample size	Age	Country	Year of experimental protocol	Fasting hours	Study design	Supervision by their coaches	dietary intake	Method of measurement	Effect
**Maughan et al. [41]**	59	18.5	Tunisia	2006	14.5	Pre/post-control	Yes	Caloric intake	3-day record+interview	↔
								Proteins		↑ 7.93% at Dur-R *vs.*Bef-R
								Fat		↔
								Carbohydrate		↔
								Water intake		↔
**Meckel et al. [42]**	10	15.1	Israel	-	-	Pre/post	Yes	Caloric intake	2-day record	↔
**Güvenç [48]**	16	16	Turkey	-	-	Pre/post	Nm	Caloric intake	2-day record	↔
**Aziz et al. [43]**	10	17.5	Singapore	-	13.5	Pre/post-control	Nm	Caloric intake	3-day record	↔
								Proteins		↔
								Fat		↔
								Carbohydrate		↔
								Water intake		↔
**Aloui et al. [53]**	12	13.3	Tunisia	-	15	Pre/post	Nm	Caloric intake	3-day record	↔
**Hammouda et al. [46]**	15	17.3	Tunisia	2010	15.5	Pre/post	Yes	Caloric intake	7-day record	↓ 15.29% Dur-R *vs.*Bef-R
								Proteins		↔
								Fat		↓ 6.46% Dur-R *vs.*Bef-R
								Carbohydrate		↔
**Bouhlel et al. [49]**	10	18.5	Tunisia	-	-	Pre/post	Nm	Caloric intake	7-day record	↓ 21.30% Dur-R *vs.*Bef-R
								Water intake		↓ 18.18% Dur-R *vs.*Bef-R
**Hammouda et al. [47]**	12	17.52	Tunisia	2010	15.5	Pre/post	Nm	Caloric intake	7-day record	↓ 14.30% Dur-R *vs.*Bef-R
								Proteins		↔
								Fat		↓ 6.21% Dur-R *vs.*Bef-R
								Carbohydrate		↔
**Bouhlel et al.**	10	18.8	Tunisia	2011	15	Pre/post	Nm	Caloric intake	7-day record	↓ 3.75% Dur-R *vs.*Bef-R

**[50]**								Proteins		↓ 23.47% Dur-R *vs*.Bef-R
								Fat		↓ 19.72% Dur-R *vs*.Bef-R
								Carbohydrate		↑ 12.68% Dur-R *vs*.Bef-R
								Water intake		↓ 16.66% Dur-R *vs*.Bef-R
**Zarrouk et al. [52]**	8	17.2	Tunisia	2009	14	Pre/post	Yes	Caloric intake	3-day record+interview	↑ 18.90% Dur-R *vs*.Bef-R
								Proteins		↔
								Fat		↑ 28.84% Dur-R *vs*.Bef-R
								Carbohydrate		↔
								Water intake		↑ 18.18% Dur-R *vs*.Bef-R

NM = not mentioned; ↓ = decrease; ↔ = no-change; ↑ = increase; *vs*.= versus; Bef-R = Before Ramadan; Dur-R = During Ramadan.

Study name	Std diff in means	Standard error	Variance	Lower limit	Upper limit	Z-Value	p-Value
Lotfi, 2010 (soccer)	0.205	0.248	0.061	-0.280	0.691	0.830	0.407
Lotfi, 2010 (runners)	-0.013	0.188	0.035	-0.381	0.356	-0.067	0.947
Güvenç, 2011	-0.085	0.194	0.038	-0.465	0.295	-0.439	0.660
Aziz, 2012	0.055	0.245	0.060	-0.426	0.535	0.223	0.823
Bouhlel, 2014	-0.082	0.245	0.060	-0.563	0.399	-0.335	0.738
Bouhlel, 2016	-0.072	0.245	0.060	-0.553	0.409	-0.293	0.769
Zarrouk, 2016	-0.041	0.274	0.075	-0.578	0.496	-0.150	0.881
	-0.011	0.086	0.007	-0.180	0.158	-0.126	0.899

Std diff in means and 95% CI

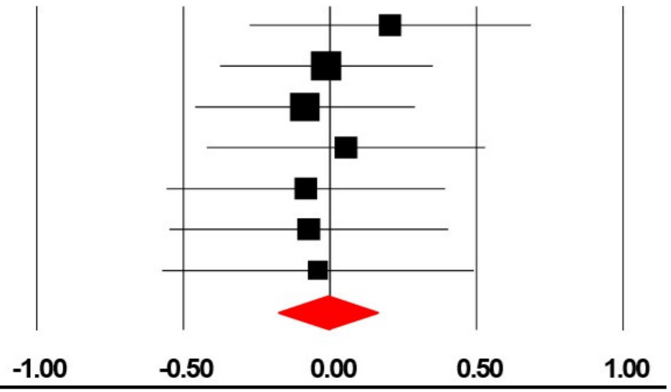

Figure S1. Forest plot for the effect of one week of Ramadan observance on body mass in adolescent athletes.

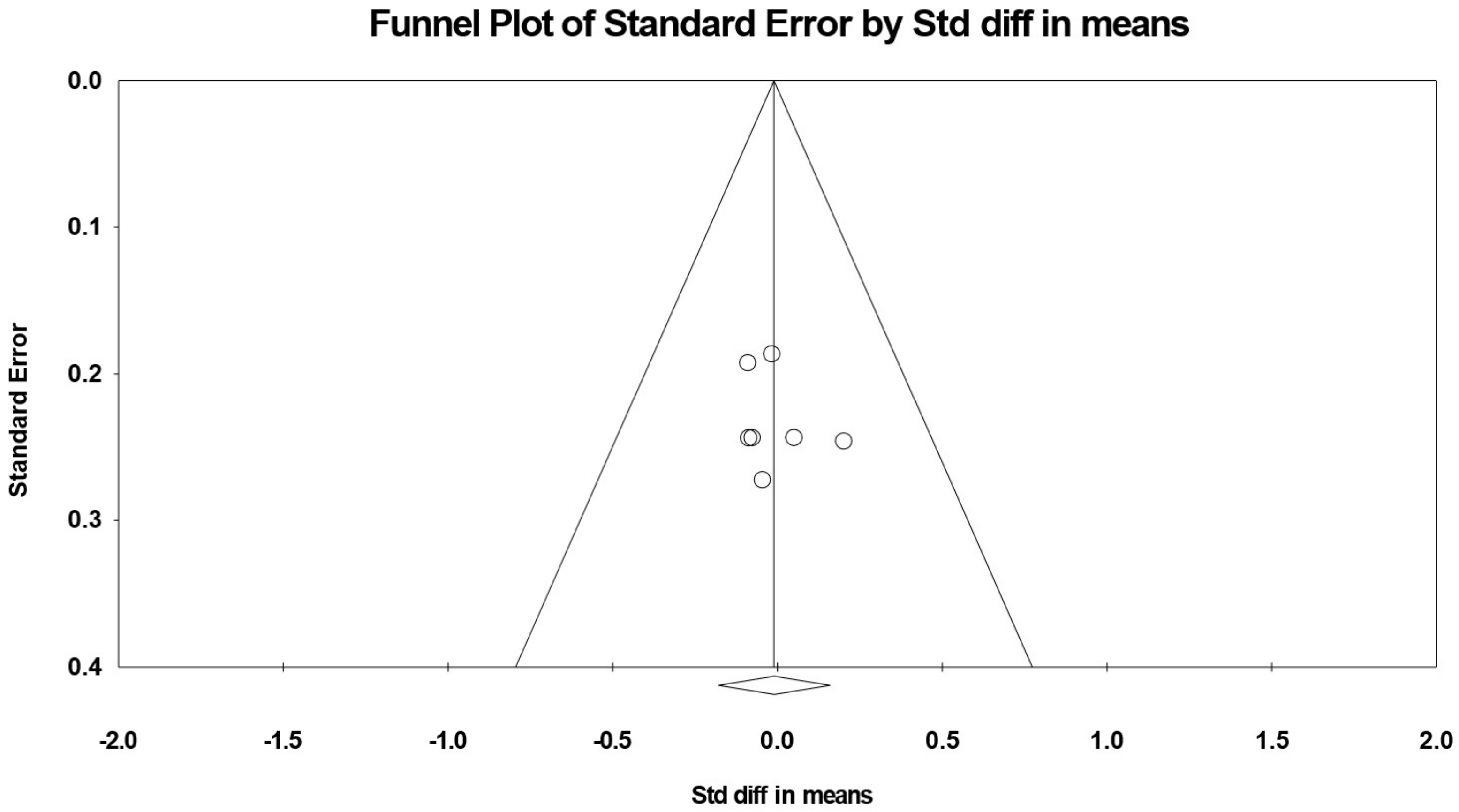

Figure S2. Funnel plot of body mass for adolescent athletes during the first week of Ramadan observance showing no evidence of publication bias.

Study name	Std diff in means	Standard error	Variance	Lower limit	Upper limit	Z-Value	p-Value
Maughan, 2008	-0.072	0.133	0.018	-0.333	0.189	-0.540	0.589
Lotfi, 2010 (soccer)	0.054	0.245	0.060	-0.426	0.534	0.221	0.825
Lotfi, 2010 (runners)	0.195	0.190	0.036	-0.176	0.567	1.031	0.303
Aziz, 2012b	-0.124	0.246	0.060	-0.606	0.358	-0.503	0.615
Hammouda, 2013	-0.120	0.201	0.040	-0.513	0.274	-0.597	0.550
Hammouda, 2014	-0.027	0.224	0.050	-0.465	0.411	-0.121	0.904
	-0.020	0.078	0.006	-0.174	0.134	-0.258	0.796

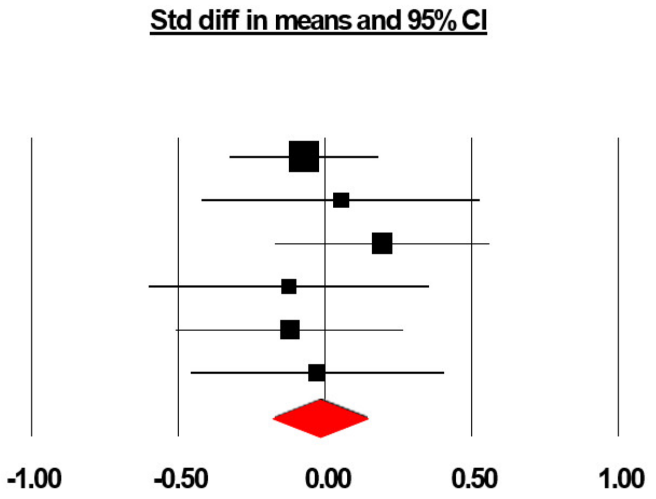

Figure S3. Forest plot for the effect of two weeks of Ramadan observance on body mass in adolescent athletes.

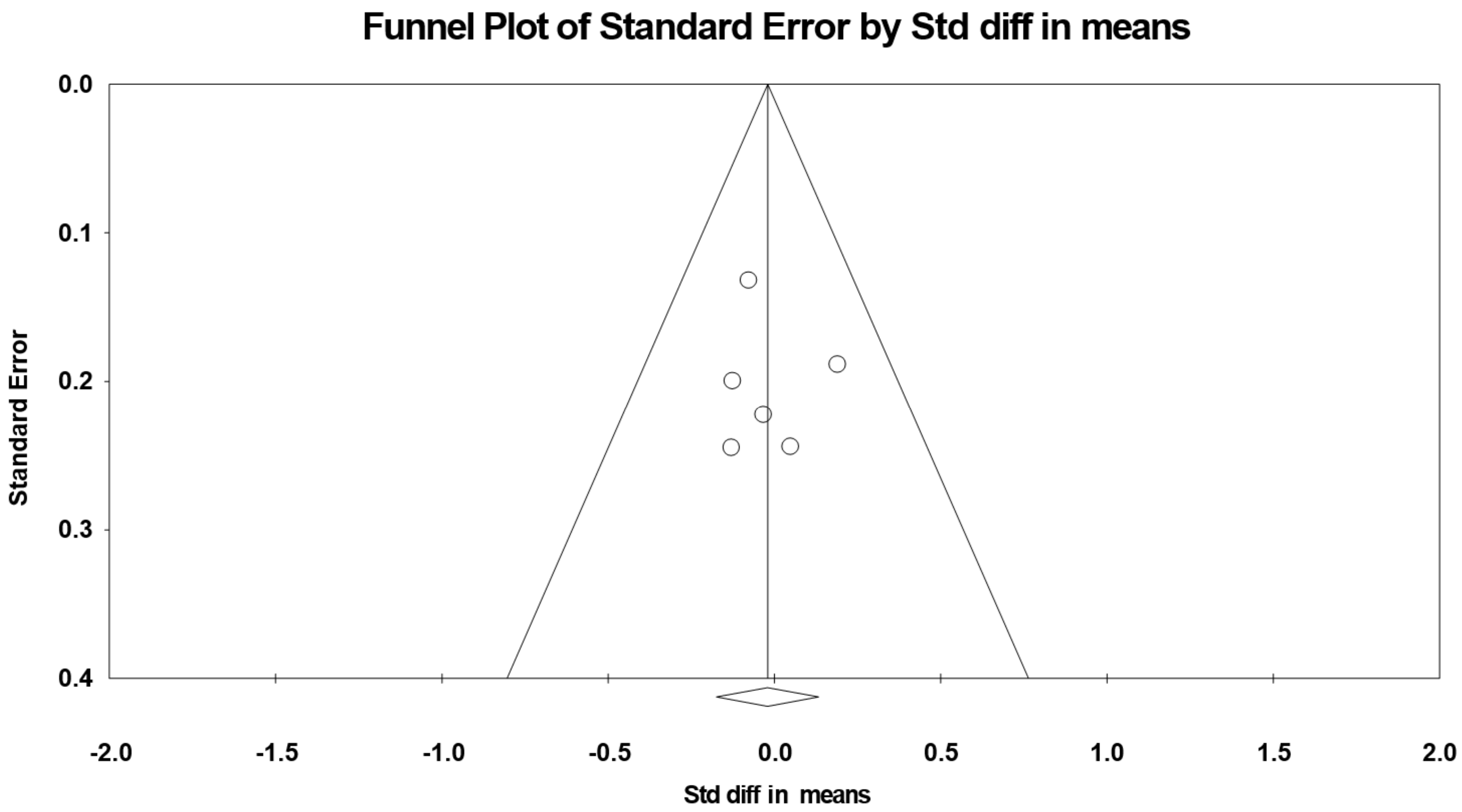

Figure S4. Funnel plot for body mass in adolescent athletes during the second week of Ramadan observance, showing no evidence of publication bias.

Study name	Std diff in means	Standard error	Variance	Lower limit	Upper limit	Z-Value	p-Value
Zarrouk, 2013	0.123	0.275	0.076	-0.416	0.662	0.447	0.655
Bouhlel, 2014	-0.031	0.245	0.060	-0.511	0.450	-0.125	0.900
Bouhlel, 2016	0.227	0.248	0.062	-0.259	0.713	0.915	0.360
	0.104	0.147	0.022	-0.184	0.393	0.707	0.480

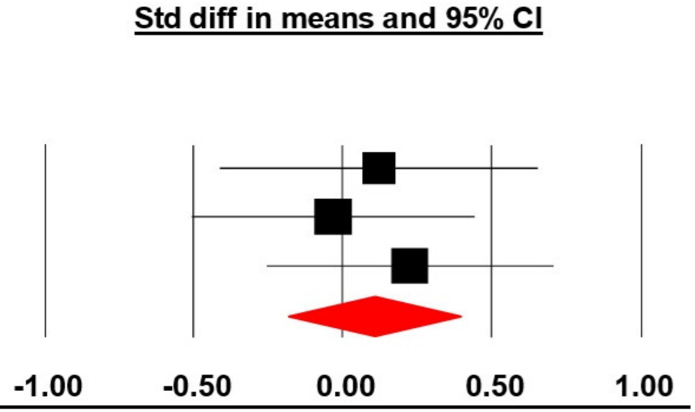

Figure S5. Forest plot for the effect of one week of Ramadan observance on body fat mass in adolescent athletes.

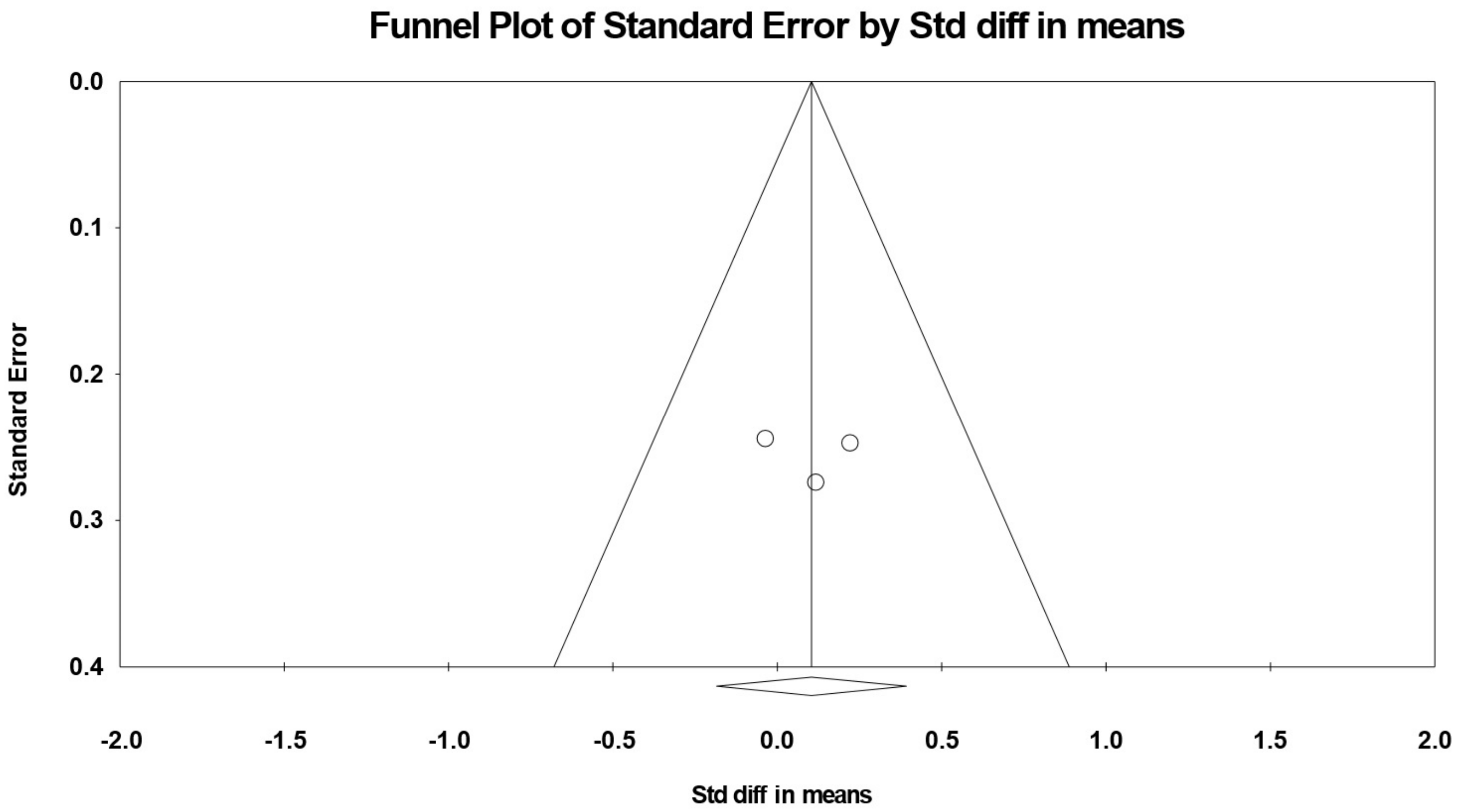

Figure S6. Funnel plot for body fat mass in adolescent athletes during the first week of Ramadan observance, showing no evidence of publication bias.

Study name	Std diff in means	Standard error	Variance	Lower limit	Upper limit	Z-Value	p-Value
Zarrouk, 2013	0.056	0.274	0.075	-0.481	0.593	0.205	0.837
Hammouda, 2014	-0.181	0.225	0.051	-0.622	0.261	-0.801	0.423
Bouhlel, 2014	-0.084	0.245	0.060	-0.565	0.396	-0.344	0.731
Bouhlel, 2016	0.344	0.252	0.064	-0.150	0.838	1.363	0.173
	0.018	0.124	0.015	-0.224	0.261	0.149	0.882

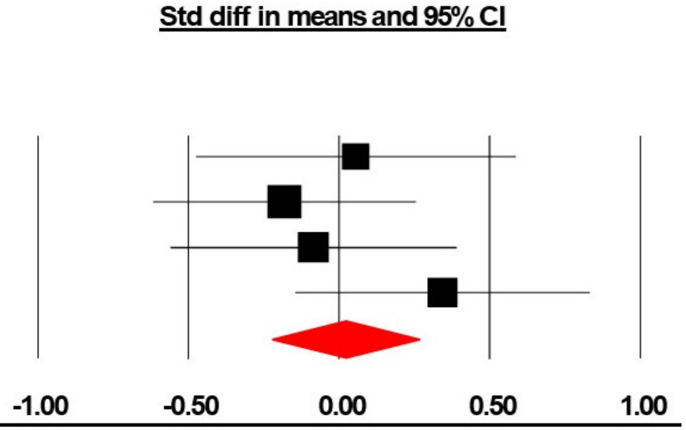

Figure S7. Forest plot for the effect of four weeks of Ramadan observance on body fat mass in adolescent athletes.

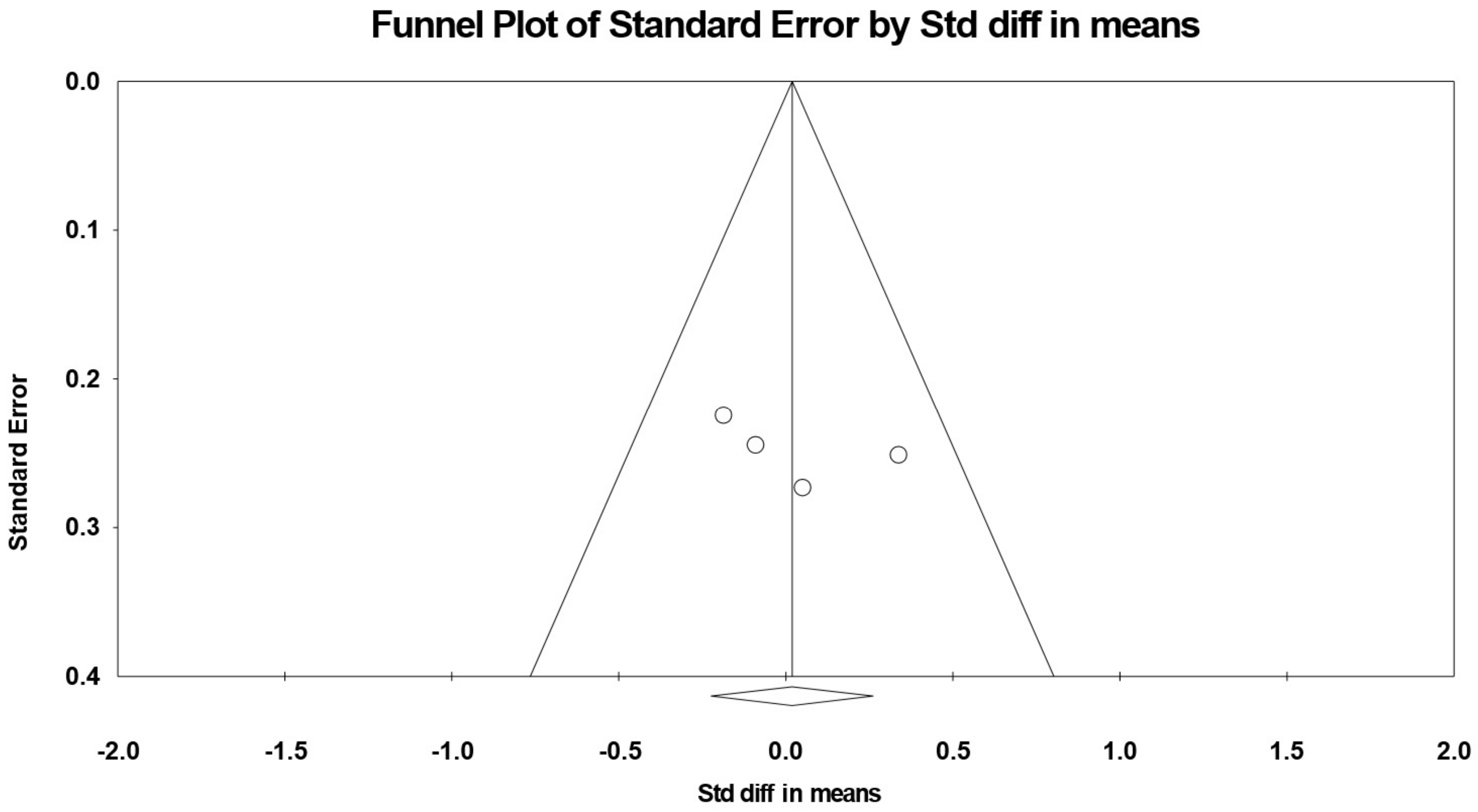

Figure S8. Funnel plot for body fat mass in adolescent athletes during the fourth week of Ramadan observance, showing no evidence of publication bias.

Study name	Std diff in means	Standard error	Variance	Lower limit	Upper limit	Z-Value	p-Value
Maughan, 2008	-0.068	0.133	0.018	-0.328	0.193	-0.509	0.611
Guvenc, 2011	-0.150	0.195	0.038	-0.532	0.232	-0.771	0.441
Hammouda, 2013	-0.091	0.200	0.040	-0.484	0.302	-0.455	0.649
	-0.093	0.096	0.009	-0.282	0.095	-0.969	0.333

Statistics for each study

Std diff in means and 95% CI

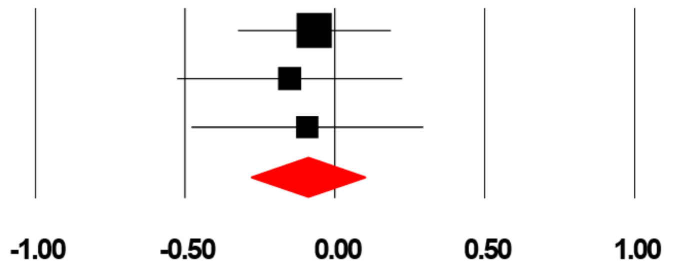

Figure S9. Forest plot for the effect of two weeks of Ramadan observance on body fat percentage in adolescent athletes.

Study name	Std diff in means	Standard error	Variance	Lower limit	Upper limit	Z-Value	p-Value
Maughan, 2008	0.031	0.133	0.018	-0.229	0.292	0.235	0.814
Hammouda, 2013	-0.027	0.200	0.040	-0.419	0.365	-0.136	0.892
	0.013	0.111	0.012	-0.204	0.230	0.121	0.904

Statistics for each study

Std diff in means and 95% CI

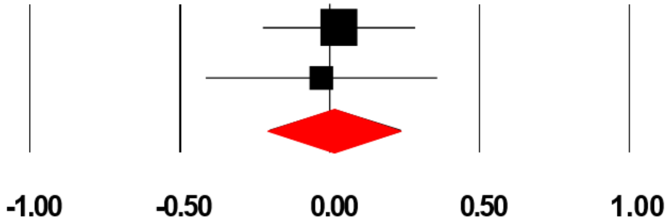

Figure S10. Forest plot for the effect of four weeks of Ramadan observance on body fat percentage in adolescent athletes.

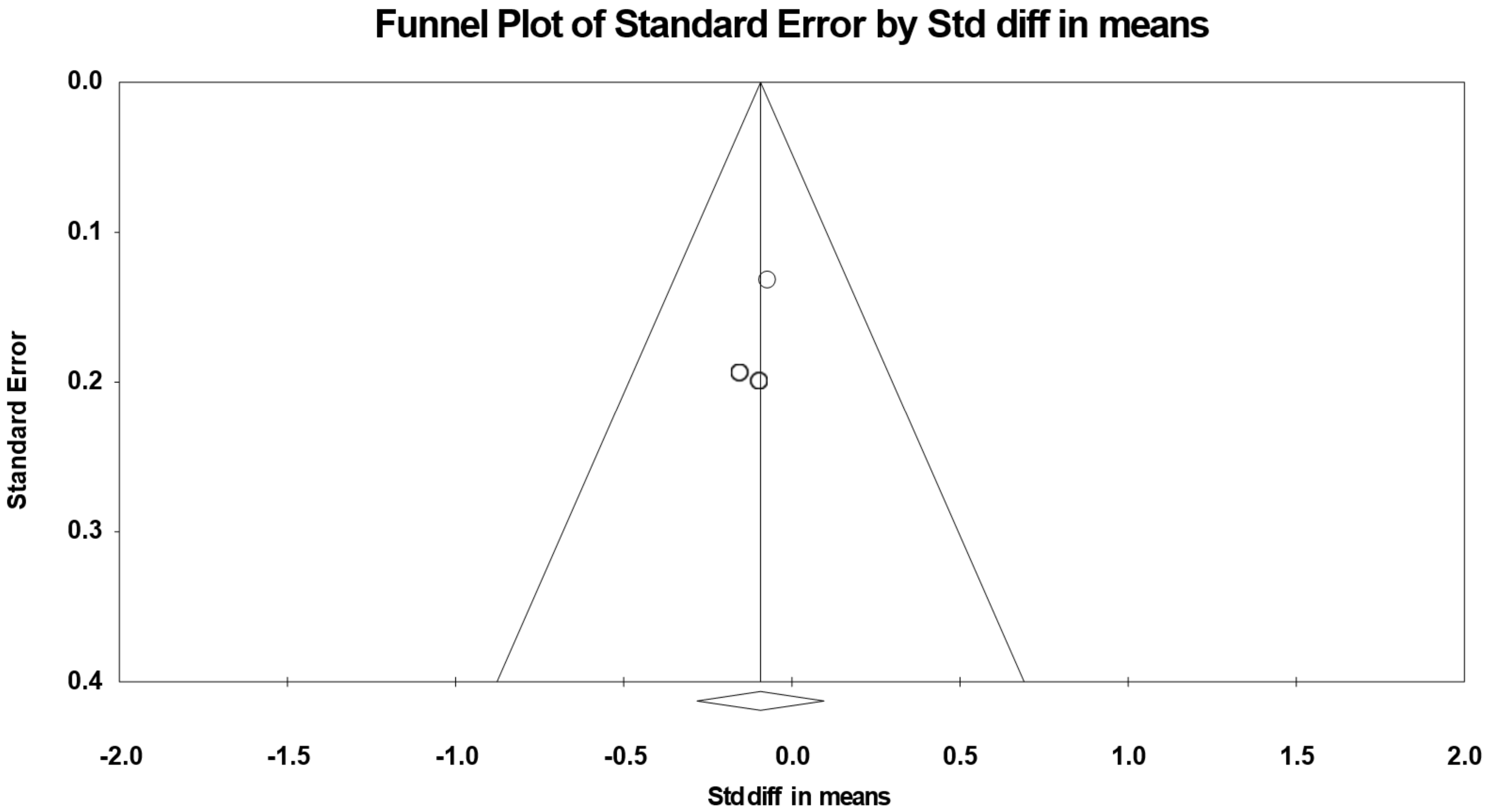

Figure S11. Funnel plot for the effect of four weeks of Ramadan observance on body fat percentage in adolescent athletes, showing no evidence of publication bias.

Study name	Std diff in means	Standard error	Variance	Lower limit	Upper limit	Z-Value	p-Value
Hammouda, 2013	0.056	0.200	0.040	-0.336	0.449	0.281	0.778
Zarrouk, 2013	-0.181	0.276	0.076	-0.722	0.361	-0.654	0.513
	-0.025	0.162	0.026	-0.343	0.292	-0.156	0.876

Std diff in means and 95% CI

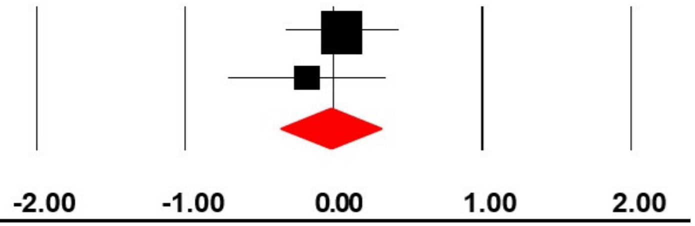

Figure S12. Forest plot for the effect of four weeks of Ramadan observance on lean mass in adolescent athletes.

Study name	Statistics for each study						
	Std diff in means	Standard error	Variance	Lower limit	Upper limit	Z-Value	p-Value
Maughan, 2008	0.399	0.105	0.011	0.194	0.605	3.811	0.000
Hammouda, 2013	0.022	0.200	0.040	-0.370	0.414	0.108	0.914
Hammouda, 2014	0.029	0.224	0.050	-0.409	0.468	0.132	0.895
Bouhlel, 2016	-0.740	0.276	0.076	-1.282	-0.199	-2.678	0.007
Zarrouk, 2016	0.521	0.292	0.085	-0.051	1.093	1.785	0.074
	0.069	0.192	0.037	-0.308	0.446	0.359	0.720

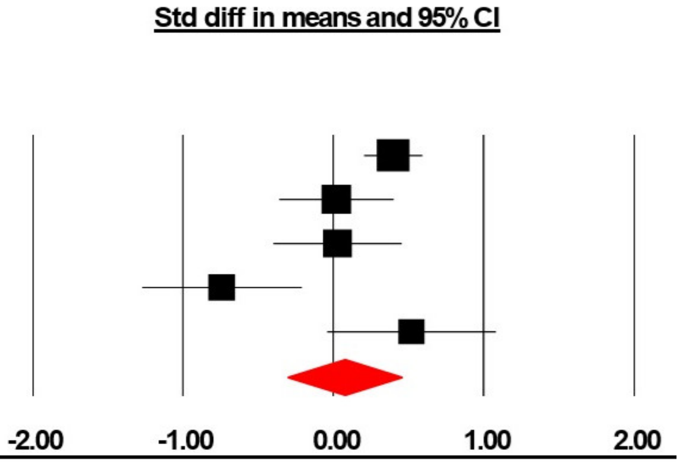

Figure S13. Forest plot for the effect of Ramadan observance on protein intake in adolescent athletes.

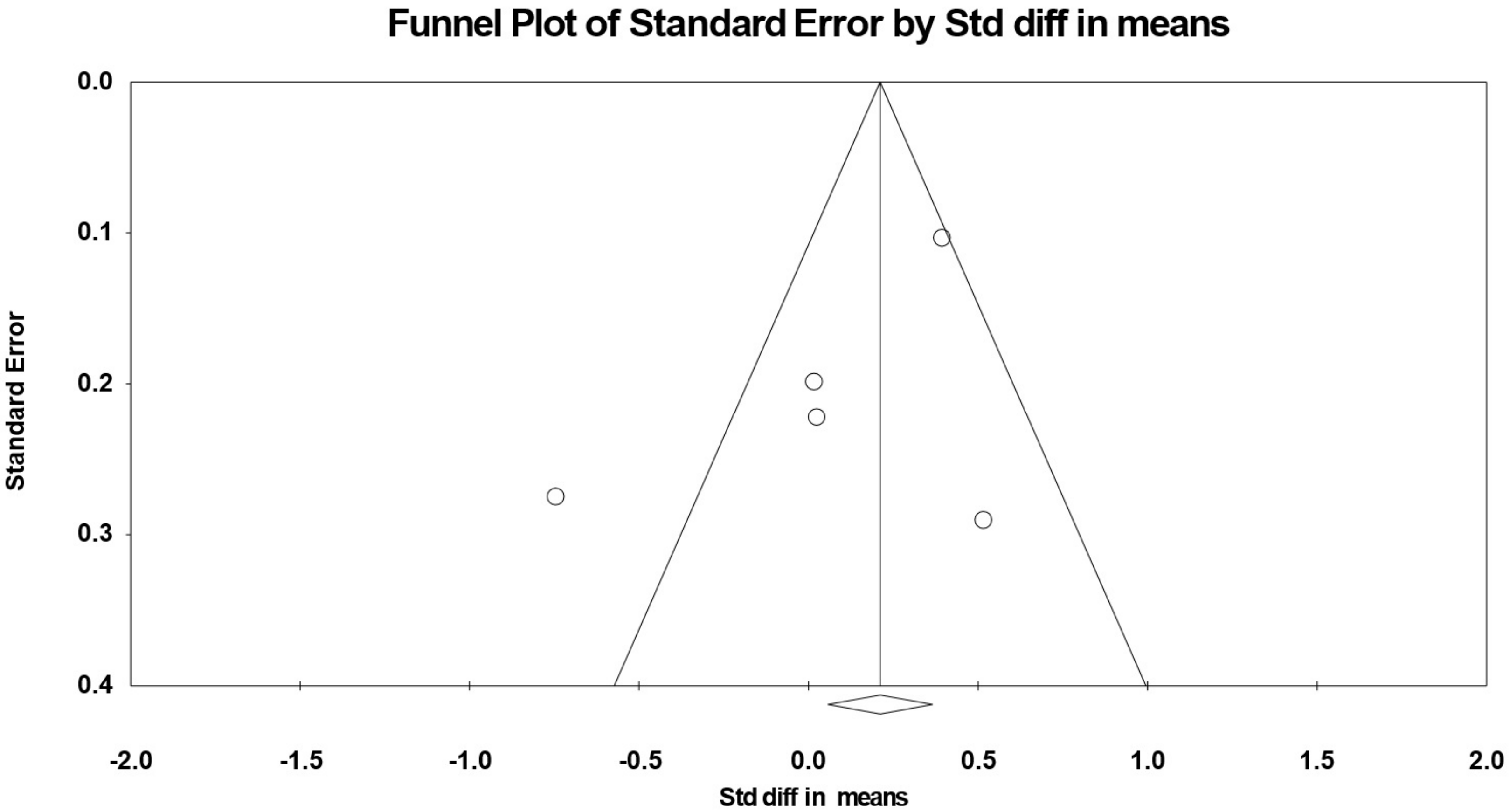

Figure S14. Funnel plot for protein intake in adolescent athletes during Ramadan observance showing no evidence of publication bias.

Study name	Statistics for each study						
	Std diff in means	Standard error	Variance	Lower limit	Upper limit	Z-Value	p-Value
Maughan, 2008	0.043	0.101	0.010	-0.154	0.241	0.429	0.668
Hammouda, 2013	-0.121	0.201	0.040	-0.515	0.272	-0.605	0.545
Hammouda, 2014	-0.111	0.224	0.050	-0.550	0.329	-0.493	0.622
Bouhlel, 2016	-0.705	0.274	0.075	-1.241	-0.168	-2.575	0.010
Zarrouk, 2016	1.529	0.403	0.163	0.739	2.320	3.791	0.000
	0.044	0.221	0.049	-0.390	0.478	0.199	0.842

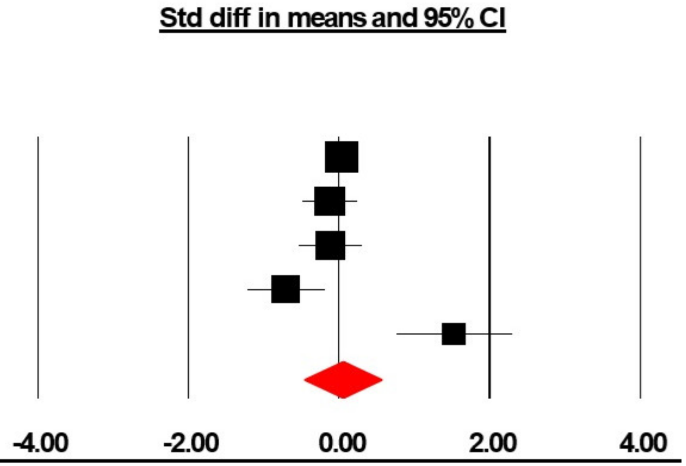

Figure S15. Forest plot for the effect of Ramadan observance on fat intakes in adolescent athletes.

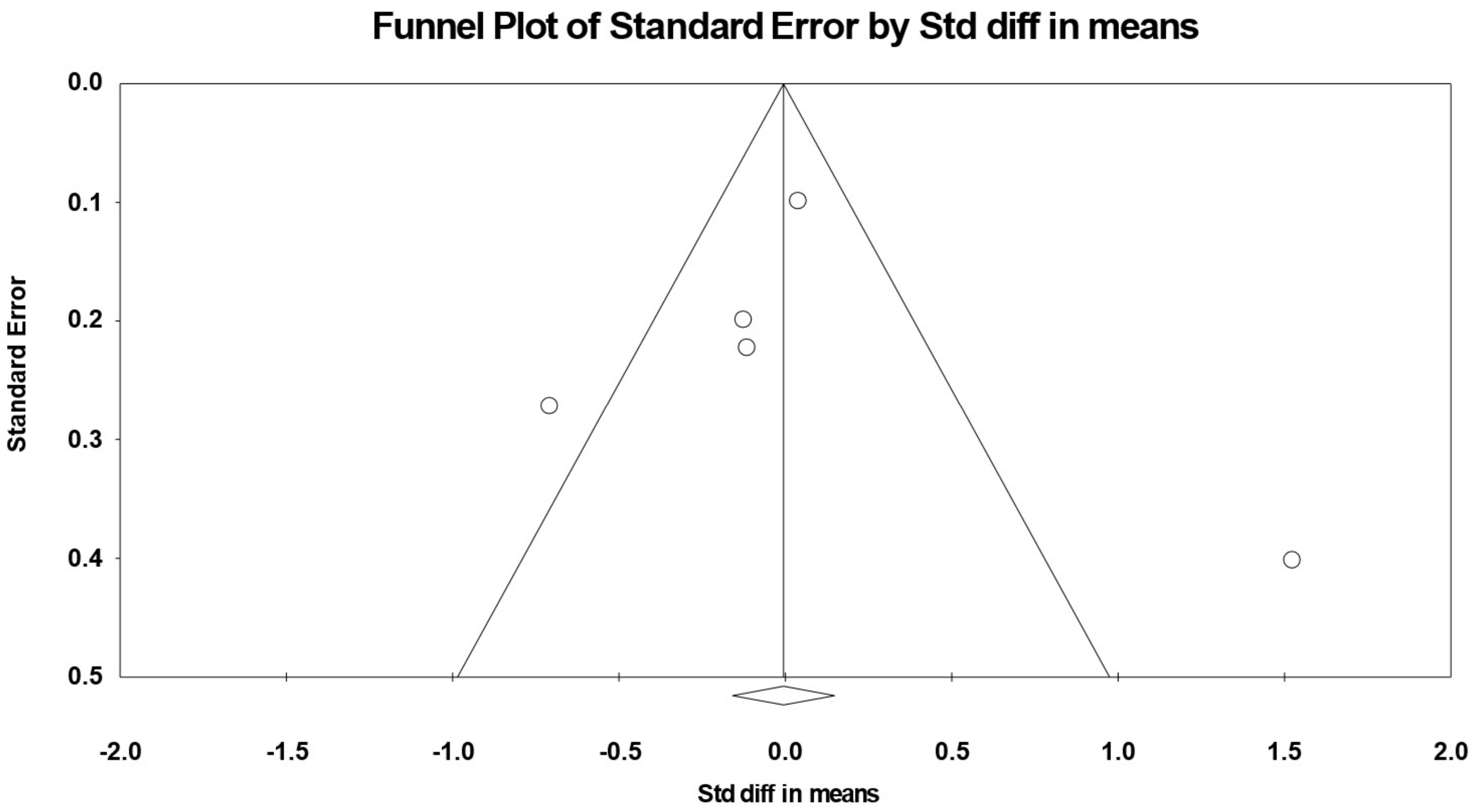

Figure S16. Funnel plot for fat intakes in adolescent athletes during Ramadan observance showing no evidence of publication bias.

Study name	Std diff in means	Standard error	Variance	Lower limit	Upper limit	Z-Value	p-Value
Maughan, 2008	-0.155	0.101	0.010	-0.354	0.043	-1.531	0.126
Hammouda, 2013	0.587	0.217	0.047	0.163	1.012	2.712	0.007
Hammouda, 2014	-0.014	0.224	0.050	-0.453	0.424	-0.064	0.949
Bouhlel, 2016	0.189	0.247	0.061	-0.295	0.673	0.764	0.445
Zarrouk, 2016	-0.151	0.275	0.076	-0.691	0.388	-0.549	0.583
	0.075	0.146	0.021	-0.211	0.362	0.516	0.606

(Statistics for each study)

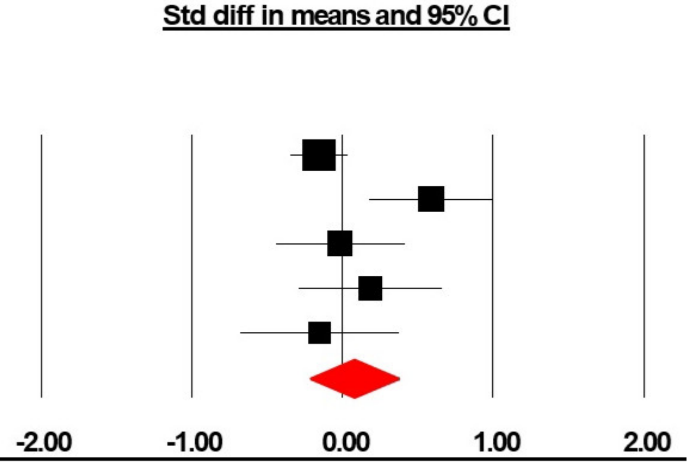

Figure S17. Forest plot for the effect of Ramadan observance on carbohydrates intakes in adolescent athletes.

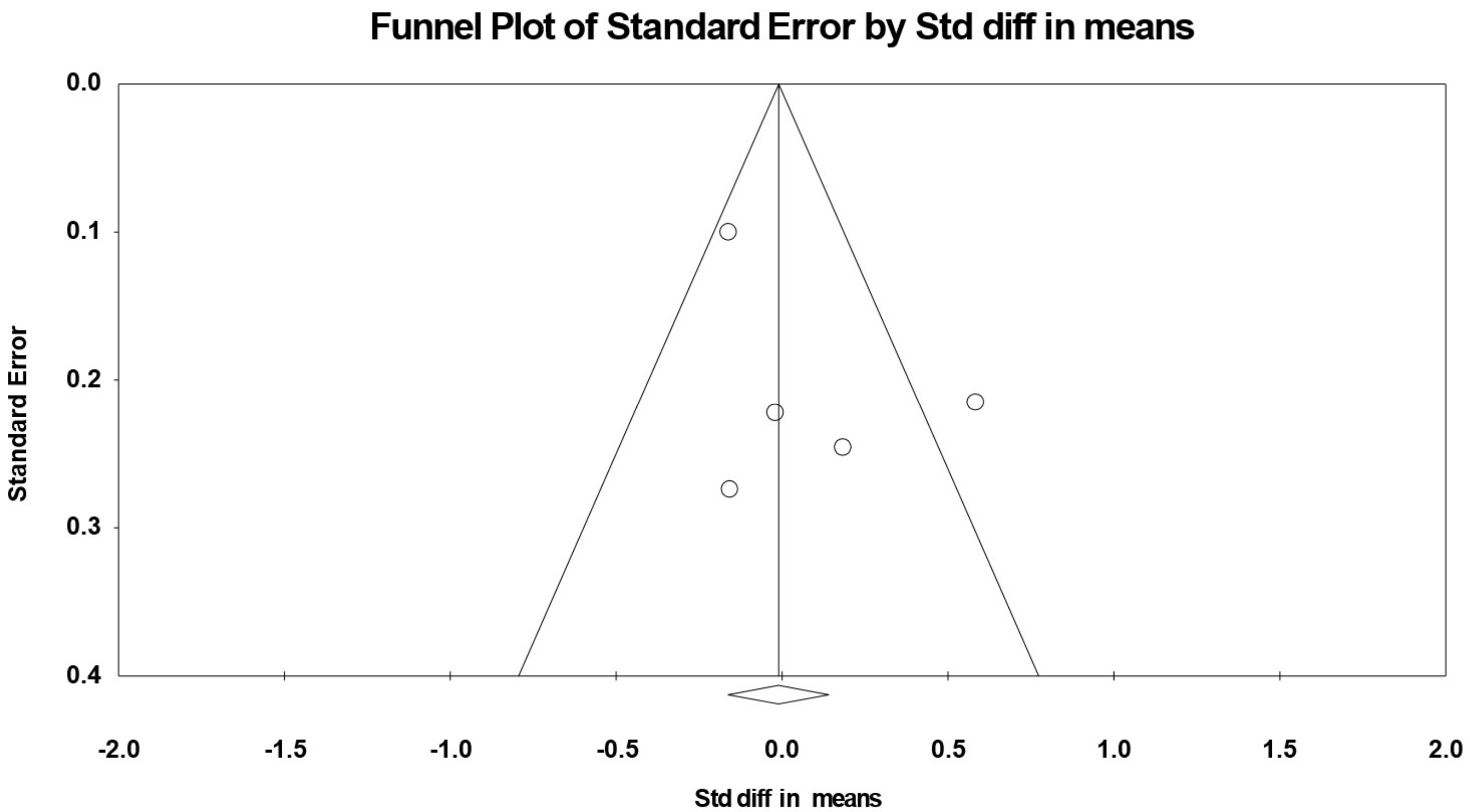

Figure S18. Funnel plot for carbohydrates intakes in adolescent athletes during Ramadan observance showing no evidence of publication bias.

Study name	Std diff in means	Standard error	Variance	Lower limit	Upper limit	Z-Value	p-Value
Maughan, 2008	-0.224	0.102	0.010	-0.424	-0.024	-2.196	0.028
Aziz, 2012	0.458	0.257	0.066	-0.047	0.963	1.779	0.075
Bouhlel, 2014	-0.667	0.271	0.073	-1.197	-0.136	-2.462	0.014
Bouhlel, 2016	-0.757	0.278	0.077	-1.301	-0.212	-2.724	0.006
Zarrouk, 2016	0.667	0.303	0.092	0.073	1.260	2.202	0.028
	-0.115	0.235	0.055	-0.576	0.346	-0.490	0.624

Statistics for each study

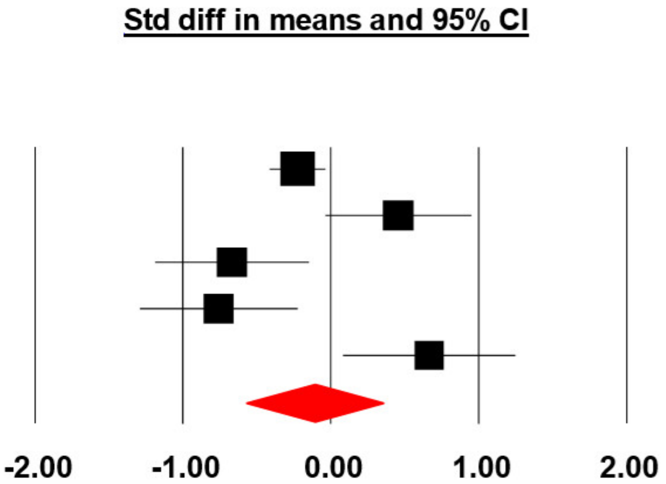

Figure S19. Forest plot for the effect of Ramadan observance on total water intakes in adolescent athletes.

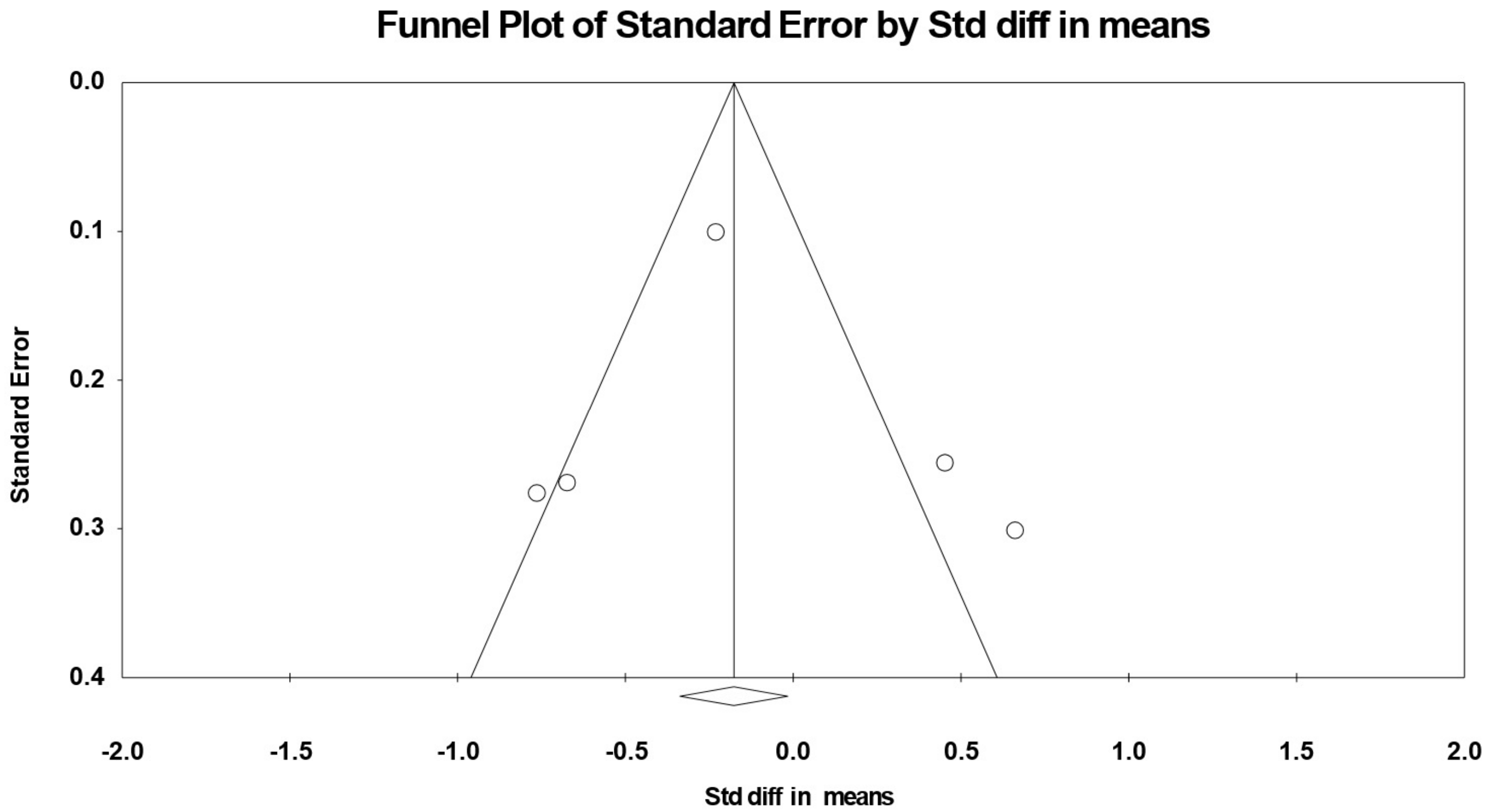

Figure S20. Funnel plot for total water intakes in adolescent athletes during Ramadan observance showing no evidence of publication bias.
